# Electronically Forbidden Raman Pathways Create a New Contrast Mechanism in Single-Molecule TERS

**DOI:** 10.1021/acs.nanolett.5c06490

**Published:** 2026-03-16

**Authors:** Hiroyuki Ikagawa, Mamoru Tamura, Hajime Ishihara

**Affiliations:** † Department of Materials Engineering Science, The University of Osaka, 1-3 Machikaneyama-cho, Toyonaka, Osaka 560-8531, Japan; ‡ Department of Physics and Astronomy, School of Science, 98311 Kwansei Gakuin University, 1 Gakuen Uegahara, Sanda, Hyogo 669-1330, Japan; ¶ Research Institute for Light-induced Acceleration System (RILACS), Osaka Metropolitan University, 1-2 Gakuencho, Nakaku, Sakai, Osaka 599-8570, Japan; § Research Organization of Science and Technology, Ritsumeikan University, 1-1-1 Nojihigashi, Kusatsu, Shiga 525-8577, Japan; ∥ SANKEN, The University of Osaka, 8-1, Mihogaoka, Ibaraki, Osaka 567-0047, Japan; ⊥ Ritsumeikan Semiconductor Application Research Center (RISA), Ritsumeikan University, 1-1-1 Nojihigashi, Kusatsu, Shiga 525-8577, Japan

**Keywords:** tip-enhanced Raman scattering, forbidden electronic transitions, nonlocal electrodynamics, vibronic coupling, multipolar Raman scattering

## Abstract

Tip-enhanced Raman scattering (TERS) provides vibrational fingerprints with subnanometer resolution, yet resonant vibronic Raman pathways involving electronically forbidden (dark) intermediate states lack a validated, fully electrodynamic description. We develop a nonlocal quantum–electromagnetic framework based on transition-dipole densities that unifies Franck–Condon and Herzberg–Teller Raman amplitudes, including their interference, and treats the Stokes frequency near field self-consistently via dyadic Green functions evaluated with a discrete-dipole approximation for arbitrary lossy plasmonic geometries. The theory shows that dark intermediate resonances can make Herzberg–Teller-derived parity information and mode-dependent nodal patterns emerge in single-molecule TERS images, enabling clear discrimination of vibrational identity and parity even under strong resonance. It also predicts that neglecting Stokes frequency backaction/renormalization (prescribed near field) can alter apparent image symmetry.

## Graphical abstract

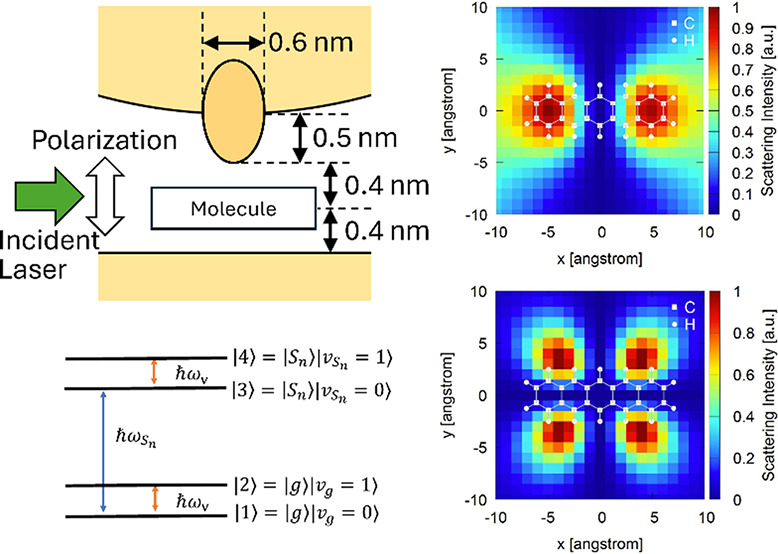

Raman spectroscopy provides a powerful window into molecular structure, yet its intrinsically weak cross section imposes severe limits on sensitivity. Surface-enhanced Raman scattering (SERS) overcomes this challenge through plasmonically amplified near fields, enabling even single-molecule detection.^1–10^ However, high sensitivity alone does not guarantee molecular-scale insight. In conventional SERS, the spatial information needed for intramolecular real-space imaging remains bounded by optical diffraction.

Tip-enhanced Raman scattering (TERS) breaks this barrier.^11–15^ Under ultra-high-vacuum and cryogenic conditions, angstrom-scale vibrational imaging has been achieved, enabling direct visualization of intramolecular modes.^16–19^ Recent single-molecule optical mapping studies have further demonstrated vibronic-state imaging and resonance-specific contrast in related systems.^20–22^ At such extreme confinement, near-field gradients can violate conventional Raman selection rules, yielding signals from nominally Raman-inactive modes.^9,23–25^ Even more strikingly, near fields can push beyond vibrational selection rules. Single-molecule studies indicate that electronic excitation selection rules can also break down. In particular, SERS experiments on single-walled carbon nanotubes revealed resonant Raman responses mediated by electronically forbidden transitions.^26^

This development exposes a decisive missing piece in our understanding of single-molecule TERS. If Raman scattering proceeds through an electronically forbidden resonant intermediate state, what determines the real-space contrast that ultimately appears in submolecular maps? In particular, it is not known whether forbidden resonances merely provide an additional weak channel or whether they can fundamentally reorganize vibronic pathways by reshaping the interference between Franck–Condon (FC) and Herzberg–Teller (HT) contributions, thereby changing the symmetry and nodal structure of the images. Because our mode discrimination is encoded in parity and nodal patterns, getting this point wrong is not a minor quantitative error. It risks a qualitatively incorrect assignment of vibrational contrast.

A number of theoretical approaches have already moved beyond the long-wavelength approximation (LWA) to address TERS in the regime where the spatial extent of the enhanced near field becomes comparable to intramolecular wave functions and a microscopic description of molecule–field coupling is required. These include extensions of Albrecht’s resonance Raman theory to near fields,^27–30^ hybrid atomistic electrodynamics–quantum mechanical frameworks,^31–34^ models formulated in terms of local electronic density,^35^ and approaches based on atomistic Raman polarizabilities.^36^ These theories reproduce key experimental trends and clarify how field gradients, molecular orientation, and tip position modify Raman selection rules in nanogaps. The role of vibronic coupling in electronically forbidden transitions has also been discussed within real-time time-dependent density functional theory using a multipole Hamiltonian.^37^

However, a central requirement for the question posed above is still missing. One must treat resonant vibronic pathways in a genuinely nonlocal manner using spatially distributed transition-dipole densities and, at the same time, describe the Stokes frequency electromagnetic response self-consistently in the spontaneous emission process (including multipolar channels), rather than prescribing the scattered field. To the best of our knowledge, existing TERS theories have not yet combined these two ingredients within a single unified framework, which is essential for predicting and interpreting submolecular images mediated by electronically forbidden resonances.

To meet this central requirement, we develop a nonlocal quantum–electromagnetic framework for TERS that is designed to capture forbidden-resonance Raman scattering on the same footing as allowed pathways. Our approach rests on three pillars: (i) a transition-dipole-density formulation that treats the electronic transition polarization as a spatially distributed quantity (beyond the LWA), retaining FC/HT vibronic Raman pathways and their interference even for electronically forbidden intermediate resonances; (ii) a fully self-consistent electrodynamic treatment of the Stokes frequency response via the Green tensor (including loss and all modes) so that the scattered field is not prescribed but renormalized by the nanogap; and (iii) an explicit treatment of multipolar light–matter couplings in strongly inhomogeneous near fields. A key consequence is that the map symmetry itself can be controlled by the FC/HT interplay together with the Stokes side backaction. Simplified descriptions that prescribe the scattered field can therefore yield qualitatively incorrect submolecular symmetries.

Using a pentacene molecule missing the two hydrogen atoms at its center (deficient pentacene) as an experimentally benchmarked system,^19^ we obtain results in very good agreement with experiment. We then show that for a normal pentacene molecule electronically forbidden resonances open a previously inaccessible contrast channel. Vibrational modes that appear to be nearly indistinguishable under electronically allowed resonances become clearly resolvable. In particular, the parity information encoded in HT-type transition-dipole densities is only ambiguously reflected under allowed resonances, whereas it can be cleanly imprinted in submolecular maps under forbidden resonances. These results indicate that electronically forbidden transitions provide a powerful new degree of freedom for mode-selective single-molecule imaging.

We develop a nonlocal quantum–electrodynamical theory of TERS in which the molecular electronic and vibrational degrees of freedom are treated quantum mechanically and the plasmonic junction is incorporated through electrodynamics. At the incident and Stokes frequencies, we treat the electromagnetic response of the same junction self-consistently together with the nonlocal molecular polarization. In the excitation step, this yields the driven near field under laser illumination, while in the emission step, it yields the renormalized dyadic Green tensor that governs spontaneous emission. The key point is that the same nonlocal transition-dipole-density description is used consistently for both excitation and emission, so that FC/HT interference and the resulting map symmetry are evaluated without prescribing the scattered field.

## Excitation (incident frequency)

We describe the molecular response using a standard real-space form of the microscopic linear susceptibility, commonly used in transition-dipole-density-based nonlocal response theories^38–40^

$$P\left(r,\omega \right)=\varepsilon_{0}\int \;dr^{\prime }\chi \left(r,r^{\prime },\omega \right)E\left(r^{\prime },\omega \right)$$ 1

where **χ**(**r**, **r′**, ω) treats the electronic transition polarization as a spatially distributed quantity beyond the LWA, thereby capturing multipolar couplings to a strongly inhomogeneous near field. With the incident laser regarded as a classical coherent drive, the tip-enhanced near field at the incident frequency follows from the self-consistent solution of Maxwell’s equations coupled to eq 1 (with the metal described by its dielectric function), yielding tip position-dependent excitation amplitudes, including gradient and multipolar effects.

## Spontaneous Emission (Stokes frequency)

Raman-scattered photons emitted from the populated vibronic level experience Purcell enhancement and additional gradient effects in the nanogap.^41,42^ At the Stokes frequency, we do not truncate the plasmonic response to a few (quasi-)­normal modes. Instead, we use the renormalized dyadic Green tensor **G**_ren_(**r**, **r′**, ω) of the full junction, defined as the solution of the corresponding Maxwell equation with the (generally complex) metal dielectric function and therefore implicitly containing the full continuum of modes (including loss) (see eqs S31 and S32 of the Supporting Information (section 1.3)).^43–45^ For the sake of completeness, we note that **G**_ren_ admits a formal spectral representation (see eq S31). In the present work, however, **G**_ren_ is evaluated numerically for the actual junction geometry, so that material absorption and radiative leakage are incorporated self-consistently without introducing phenomenological line widths or a discrete-mode truncation. This Green tensor formulation has also been employed in nonresonant SERS, and here we show that it can be combined consistently with resonant Raman expressions in TERS.^46^

Assuming that the molecule is initially in its ground state, the Raman photon flux (or intensity) at scattered frequency ω_s_ can be written as

S(ωs)=1π∑n,mRe[∑p=14Dn,m(p)(ωi,ωs)Yn,m(p)(ωs)In,m(p)(ωi)] 2

where *D*_*n*,*m*_^(*p*)^ terms are molecular energy denominators and *I*_*n*,*m*_^(*p*)^ and *Y*_*n*,*m*_^(*p*)^ encode the excitation and Stokes side emission processes through the incident near field and renormalized Green tensor, respectively. The four contributions arise from the two Kramers–Heisenberg (absorption-first/emission-first) terms and their interference.^47^ Explicit expressions for *D*_*n*,*m*_^(*p*)^, *I*_*n*,*m*_^(*p*)^, and *Y*_*n*,*m*_^(*p*)^ are given in section 1.3 of the Supporting Information. Within the present formulation, eq 2 is used without an explicit treatment of finite-temperature/environmental effects or population-driven emission. These points, together with remarks on stronger plasmon–molecule backaction and model scope, are discussed briefly in section 2 of the Supporting Information.

Furthermore, in the nonresonant Raman regime, eq 2 can be further approximated to provide a simpler description of the intermediate states. The corresponding derivation and approximate expression are given in section 1.6 of the Supporting Information. In the following, whenever we refer to nonresonant Raman scattering, we use this approximate form in our calculations.

Our calculation model is shown in Figure 1a. The tip apex is modeled as a gold nanosphere with an atomistic protrusion positioned above a gold substrate with the target molecule in the gap. The atomistic structure commonly associated with angstrom-scale resolution is shown in Figure 1b.^48^ In experiments, a thin NaCl layer is often inserted to electronically decouple the molecule from the metallic substrate and suppress hybridization/charge transfer.^18^ In our model, this effect is captured at an effective level by introducing a finite tip–substrate separation (and the corresponding dielectric gap), which reduces direct metal–molecule hybridization while keeping the electrodynamic response of the junction. Under this electronically decoupled condition, conductive/tunneling charge transfer across the junction, and thus charge-transfer plasmon effects, are expected to be strongly suppressed and are neglected in the present calculations. The molecular electronic structure is reduced to an effective four-level scheme (Figure 1c) within the Born–Oppenheimer approximation.^49^

**Figure 1:**
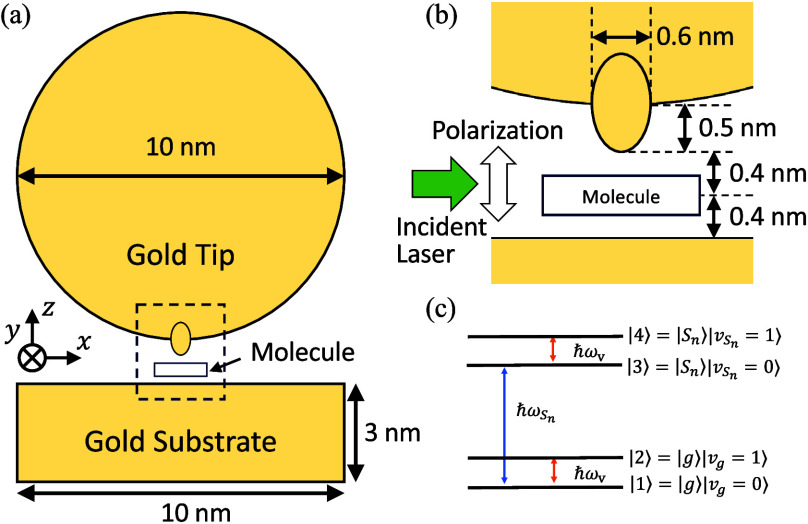
(a) Schematic of the TERS calculation model. (b) Detailed atomic configuration of the picocavity. (c) Effective four-level molecular model used in the calculations. ω_S_*n*__ and ω_v_ represent electronic excitation energy for the S_*n*_ excited state and vibrational frequency, respectively.

As benchmark systems, we employ pristine pentacene and deficient pentacene.^19^ Electronic-structure calculations and vibrational analyses were performed using Gaussian 16.^50^ Figure 2 shows the relevant molecular orbitals of pentacene and the spatial distributions of the FC transition-dipole densities for states S_1_ and S_2_. Specifically, panels a–d show the highest occupied molecular orbital-1 (HOMO–1), HOMO, lowest unoccupied molecular orbital (LUMO), and LUMO+1 of pentacene, respectively, while panels e and f display the FC transition-dipole densities for states S_1_ and S_2_, respectively. The S_1_ excitation is dominated by the HOMO–LUMO transition and is optically allowed, with a finite transition dipole along the molecular short axis. In contrast, S_2_ is mainly composed of the HOMO–1–LUMO and HOMO–LUMO+1 configurations, which connect orbitals of the same parity and yield a transition-dipole density that cancels upon integration over the molecule, rendering S_2_ electronically forbidden.

**Figure 2:**
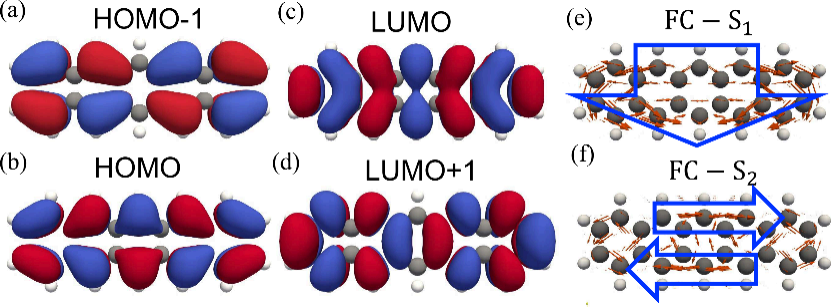
(a–d) Electronic molecular orbitals of pentacene (HOMO–1, HOMO, LUMO, and LUMO+1, respectively). (e and f) Spatial distributions of the FC terms of transition-dipole densities for states S_1_ and S_2_, respectively. The orange arrows represent the transition-dipole moment density at each point, while the blue arrows indicate the overall orientation of the transition-dipole moment.

We also evaluate the HT contributions to the transition-dipole densities for each vibrational mode by incorporating vibronic coupling within a linear vibronic coupling (Herzberg–Teller) treatment.^49,51,52^ FC overlaps are computed using the linear coupling model and the FCclasses3 package.^53–55^

The electromagnetic response of the picocavity is obtained using the discrete-dipole approximation (DDA),^56^ which we use to compute both the driven near field (incident frequency) and the renormalized Green tensor for the same lossy junction geometry. TERS images are generated by scanning the lateral tip position in the *x–y* plane and evaluating the Raman intensity mediated by either the allowed S_1_ or the forbidden S_2_ intermediate resonance. Further details—including the quantum-chemical calculations, transition-dipole densities, model parameters (molecule and DDA), and orbitals/transition-dipole densities of deficient pentacene—are provided in section 3 of the Supporting Information. Although we focus on the contrast mechanism and map symmetry using normalized intensities, we provide non-normalized TERS signal levels and an order of magnitude detectability estimate in section 4 of the Supporting Information.

We first validate our nonlocal quantum–electromagnetic framework by comparing calculated TERS images for hydrogen-deficient pentacene with previous single-molecule experiments.^19^ Here, the purpose of the comparison is not a quantitative reproduction of absolute intensities but a benchmark of the symmetry and nodal structure of submolecular maps that are governed primarily by the vibrational displacement patterns and the associated transition-dipole densities. Although the experiment in ref 19 was performed under off-resonant conditions, we find that the qualitative map symmetry/pattern is robust against detuning (while the absolute signal level changes); an explicit resonant/off-resonant comparison for the same junction geometry is provided in section 4 of the Supporting Information. We also note that the experimentally studied molecule is directly adsorbed on the metal substrate, whereas our DFT model neglects explicit molecule–substrate hybridization; accordingly, the present comparison should be regarded as a symmetry-level validation.

We now describe the calculated results for nonresonant TERS. In the previous study by Xu and co-workers, the excitation energy of the target molecule was estimated to be close to 1.7 eV whereas an excitation wavelength of 532 nm (approximately 2.33 eV) was used.^19^ To perform calculations under comparable detuning, we assumed an incident photon energy of 2.6 eV for a molecular excitation energy of approximately 2.0 eV in our model. We focus on four vibrational modes, v1–v4, with wavenumbers of 239, 381, 460, and 496 cm^–1^, respectively, which correspond to experimentally reported modes at 256, 340, 454, and 474 cm^–1^, respectively.^19^ The vibrational displacement patterns of these modes are shown in panels a–d, respectively, of Figure 3, and the corresponding HT transition-dipole densities and nonresonant TERS images are displayed in panels e–h and i–l, respectively. (We emphasize that panels a–h visualize vector fields. In each panel, the arrow direction indicates the local vector direction, and the arrow length is proportional to the local magnitude.)

**Figure 3:**
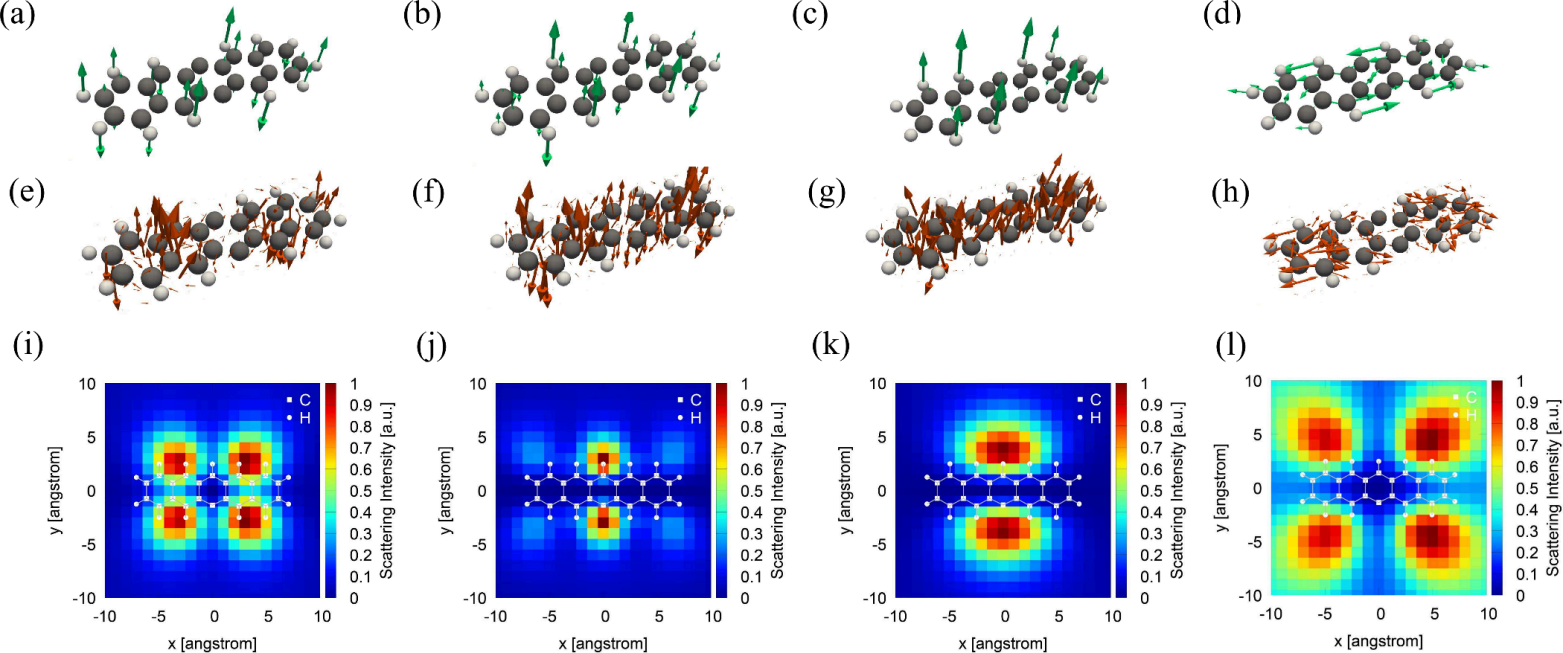
(a–d) Vibrational displacement patterns of deficient pentacene for modes v1–v4, respectively. (e–h) Spatial distributions of the HT contributions to the transition-dipole moment for S_1_, corresponding to v1–v4, respectively. (i–l) Calculated nonresonant TERS images for v1–v4, respectively. The vibrational wavenumbers are 239, 381, 460, and 496 cm^–1^, respectively.

The nonresonant TERS maps for v2 and v3 (panels j and k, respectively, of Figure 3) display two regions of enhanced scattering near the molecular center, separated along the short molecular axis. By contrast, the TERS images for v1 and v4 (panels i and l, respectively, of Figure 3i exhibit four lobes surrounding the molecule. Modes v1 and v3 are predominantly out-of-plane vibrations, and their HT transition-dipole densities possess significant out-of-plane components. As shown in panels a–c and e–g of Figure 3, Raman scattering is strongly enhanced when the tip is positioned above regions where these out-of-plane HT components are large. Mode v4 is mainly an in-plane vibration, and its HT transition-dipole density is oriented along both the long and short molecular axes on the terminal benzene rings. When the tip is located above these terminal rings, the near field couples efficiently to the in-plane HT dipoles, resulting in strong Raman scattering. Overall, these spatial patterns reflect the symmetry and nodal structure of the underlying vibrational modes and are in qualitative agreement with the trends observed in the TERS experiments of ref 19.

In the previous simulation model reported in ref 19 (and used for comparison here), the in-plane components of the Raman polarizability were neglected. To facilitate a direct comparison with those results, we also performed calculations in which all in-plane components of the transition-dipole moments appearing in eq 2 were set to zero. The corresponding TERS image for v4 is shown in Figure S5 (section 5). Under this approximation, we find that scattering is enhanced only when the tip is positioned extremely close to the molecule, closely resembling the simulation results reported in the previous study.

By contrast, the results shown in Figure 3l exhibit strong scattering even at tip positions farther from the molecular framework, in better agreement with the experimental observations. These findings indicate that in-plane components of the transition-dipole moment cannot be neglected, particularly for TERS associated with in-plane vibrational modes.

For the sake of completeness, we also provide additional simulations for the singly dehydrogenated pentacene configuration treated in ref 19 in section 6 of the Supporting Information.

We now turn to pristine (normal) pentacene and focus on two vibrational modes with wavenumbers of 919 cm^–1^ (v5) and 1027 cm^–1^ (v6), shown in panels a and b, respectively, of Figure 4. The corresponding HT transition-dipole-density vectors for excitation via S_1_ are displayed in panels c and d of Figure 4. (Here, the arrows indicate the local transition-dipole-density vector, with direction showing the local polarization and length showing its magnitude.) The corresponding HT transition-dipole-density vectors for excitation via S_2_ are displayed in panels e and f, respectively, of Figure 4. For the v5 mode in Figure 4c, the S_1_ HT transition polarization is distributed over two regions oriented in opposite directions along the molecular long axis, with its sign reversing across the molecular center. In addition, the S_2_ HT transition dipole for v5 in Figure 4e exhibits a distribution that generates a net moment along the long axis over the entire molecule. By contrast, for the v6 mode in Figure 4d, the S_1_ HT transition dipole produces a net moment along the molecular short axis, whereas the S_2_ HT transition dipole in Figure 4f shows a distribution that gives rise to two oppositely directed moments along the short axis. Because v6 is a totally symmetric mode with a large Franck–Condon factor, the Raman scattering process can be completed via vibrational-state changes driven solely by the FC transition-dipole contribution, irrespective of the spatial distribution of the HT transition dipole.

**Figure 4:**
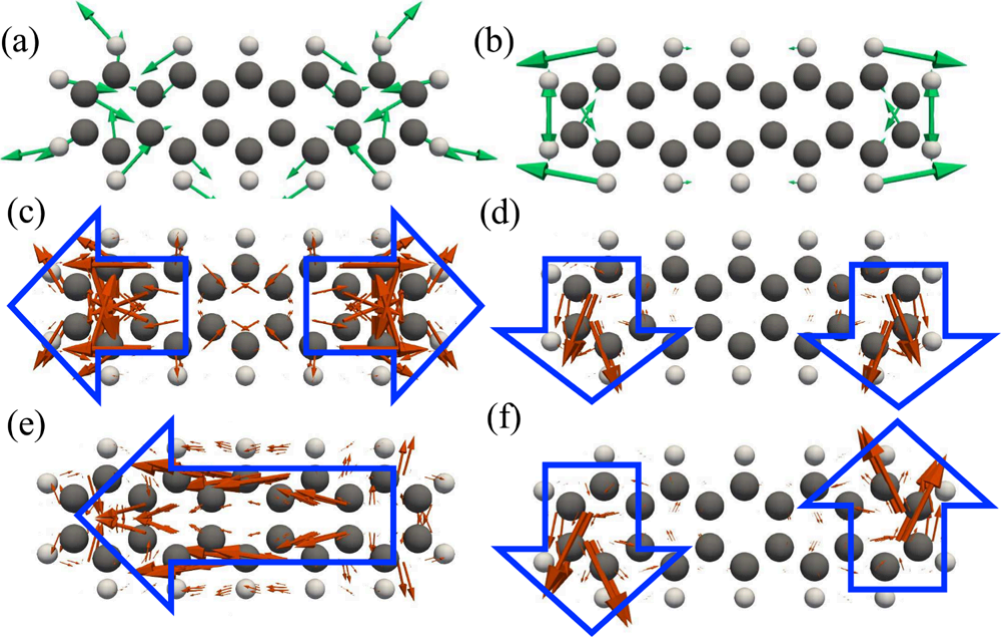
(a and b) Vibrational modes of pristine pentacene with wavenumbers of 919 cm^–1^ (v5) and 1027 cm^–1^ (v6), respectively. (c and d) HT transition-dipole-density vectors associated with the S_1_ excitation for v5 and v6, respectively. (e and f) HT transition-dipole-density vectors associated with the S_2_ excitation for v5 and v6, respectively.

Panels a and b of Figure 5 show the calculated S_1_-resonant TERS images (ω_i_ = ω_S_1__ + ω_v_) for v5 and v6, respectively. In both cases, the dominant scattering appears as two lobes separated along the molecular short axis. The TERS map for v6 in Figure 5b can be understood straightforwardly. When the tip is scanned along the short axis from the molecular center, scattering arises from the coupling between the near field and the FC transition dipole oriented along the short axis. By contrast, the v5 map in Figure 5a can be interpreted as being shaped by the combined action of an “FC background” and an HT-induced contribution within the nanogap. The v5 HT transition-dipole density shown in Figure 4c can interact most strongly with the near field when the tip is positioned at the molecular center. However, because the FC transition dipole that governs spontaneous emission also interacts with the tip-enhanced near field, the resulting pattern can be viewed as splitting into a lobe structure similar to that observed for v6. As a result, the structure of the HT transition dipole associated with S_1_ for v5 is masked in the TERS map by the contribution from the FC transition dipole. Consequently, the v5 TERS image becomes very similar to that of v6, suggesting that distinguishing them experimentally would be difficult.

**Figure 5:**
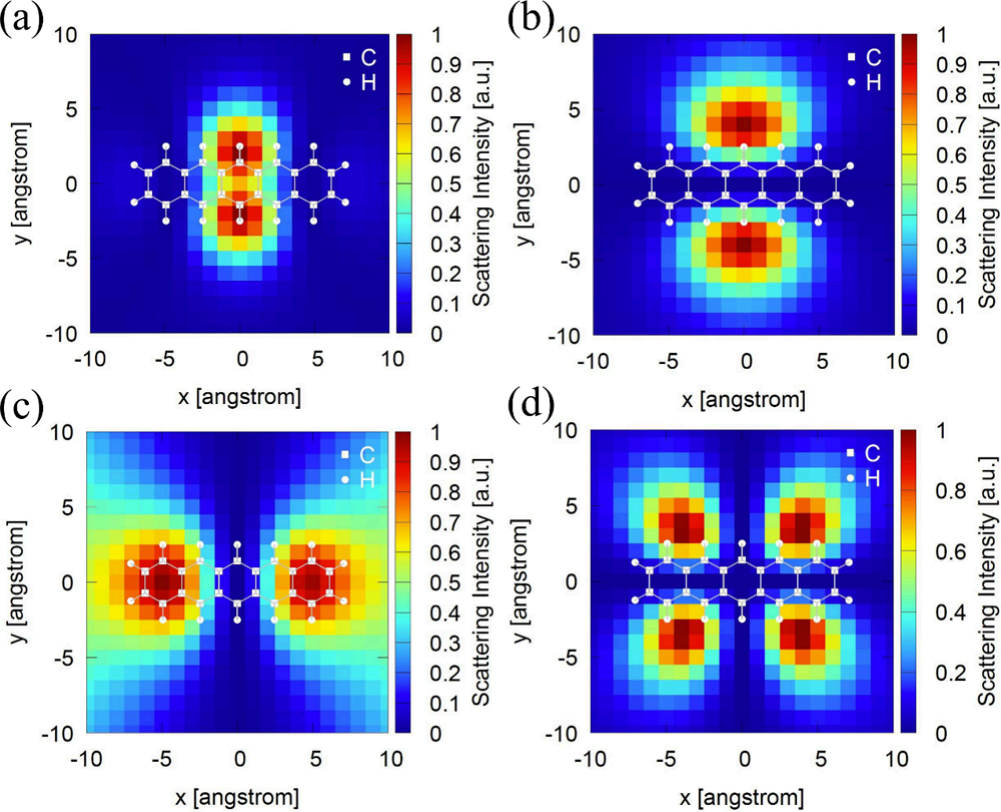
(a and b) Calculated TERS images for pristine pentacene under S_1_-resonant excitation (ω_i_ = ω_S_1__ + ω_v_) for v5 and v6, respectively. (c and d) Calculated TERS images under S_2_-resonant excitation (ω_i_ = ω_S_2__ + ω_v_) for v5 and v6, respectively.

In stark contrast, the S_2_-resonant TERS images in panels c and d of Figure 5 display qualitatively different spatial patterns and thereby separate the two modes much more clearly. Under S_2_-resonant excitation (ω_i_ = ω_S_2__ + ω_v_), v5 exhibits a two-lobe pattern split along the long molecular axis with a pronounced node around the molecular center (Figure 5c), whereas v6 shows four localized lobes around the molecular periphery (Figure 5d). The regions of maximal scattering in Figure 5c coincide with the positions where the near field interacts most strongly with the S_2_ HT transition dipole of v5, which generates a moment oriented along the molecular long axis, as shown in Figure 4e. In this sense, the TERS map can be regarded as directly reflecting the structure of the HT transition dipole. For the v6 TERS map in Figure 5d, the scattering can be understood as arising when the tip is positioned near the four molecular corners, where the multipolar FC transition dipole can partially interact with the near field. Importantly, whereas the v5 and v6 TERS maps exhibit behavior that is difficult to distinguish under S_1_-resonant excitation, they show sufficiently pronounced differences under S_2_-resonant excitation that the two modes can be clearly discriminated in real space. These results strongly suggest that vibrational selectivity beyond what can be achieved by conventional resonant TERS mediated by electronically allowed transitions can be realized through resonant TERS mediated by electronically forbidden transitions. In particular, we consider that the electronically forbidden transition interacts less strongly with the tip than an electronically allowed transition, so its influence on the TERS map is limited; this likely enables the structure of the HT transition dipole to be transferred to the TERS map with minimal masking.

Moreover, the fact that the HT transition-dipole structure associated with v5 can be directly visualized without being obscured by interference from the FC transition dipole implies that the vibrational characteristics can be examined and measured in greater detail, for example, symmetry information such as vibrational parity. For a totally symmetric vibration (v6), vibronic mixing preserves the parity class of the electronic transition-dipole density, and the HT contribution therefore tends to inherit the same symmetry character as the parent electronic excitation. In contrast, for an odd-parity (non-totally symmetric) vibration such as v5, the HT effect generates components with parity different from that of the parent electronic state. As a consequence, the HT transition dipole obeys selection rules that are the opposite of those associated with the FC transition dipole. In other words, if the FC transition dipole behaves as optically allowed, the HT transition dipole behaves as optically forbidden and vice versa. However, for Raman scattering mediated by S_1_, the FC and HT contributions can be activated in spatially overlapping regions, and their interference, including cross terms mediated by *I*_*n*,*m*_^(*p*)^(ω_i_) and *Y*_*n*,*m*_^(*p*)^(ω_s_), may obscure the underlying contrast (Figure 5a). As a result, a mode such as v5 may appear similar to a vibration with a completely different symmetry (e.g., v6). For the electronically forbidden S_2_ pathway, however, the FC background is suppressed, and the pathway selection emphasizes HT-enabled transition polarization. Consequently, the parity-driven nodal structure becomes visible directly in the real-space image (Figure 5c), and mode symmetry information can be read out from the map.

In other words, the fact that the resonant TERS map mediated by the electronically forbidden transition reveals the optically allowed character of the HT transition dipole, i.e., behavior opposite to the selection rules of the forbidden electronic transition, can serve as evidence that v5 has an odd parity. Although the present discussion involves pentacene, which has a relatively simple structure, the same considerations may be important for more general molecular systems. This is because FC/HT transition dipoles and multipolar contributions are ubiquitous in the molecular optical responses. Depending on the molecular species, a wide variety of FC–HT interference effects can arise and observations exploiting forbidden excitations may likewise become feasible.

The symmetry-selective contrast mechanism identified here is revealed only when the Stokes side response of the junction is treated self-consistently within our nonlocal framework. A direct control comparison that makes this point unambiguous, benchmarking against a non-self-consistent reference model without Stokes side backaction/renormalization, is provided in section 7 of the Supporting Information.

Experimentally, the present contrast mechanism is expected to be observable under conditions where (i) the excitation energy is tuned close to an electronically forbidden intermediate resonance, (ii) the junction field is confined on the subnanometer scale so that nonlocal (multipolar) couplings are activated, and (iii) vibronic (HT-enabled) pathways contribute appreciably to the resonant Raman amplitude and its interference with the FC background. Such conditions are consistent with recent single-molecule TERS experiments in picocavities operating under cryogenic and ultra-high-vacuum environments.

These results suggest that TERS measurements mediated by electronically forbidden transitions provide a powerful, symmetry-selective readout of vibrational modes, enabling one to access parity and nodal information directly from submolecular Raman maps.

In summary, we have established a nonlocal quantum–electromagnetic theory of TERS that consistently treats Raman scattering through electronically allowed and forbidden transitions. This framework incorporates spatially distributed multipolar transition-dipole densities and their nonlocal coupling to the tip-enhanced near field in the excitation and Stokes side emission steps. It yields results in good agreement with experimental observations and further shows that not only out-of-plane but also in-plane transition-dipole components can be essential for reproducing realistic TERS maps.

We also showed that when mode discrimination and symmetry assignment are ambiguous under resonant scattering mediated by electronically allowed transitions, the same modes can become clearly distinguishable when the Raman process proceeds via an electronically forbidden intermediate resonance. This symmetry-selective contrast emerges from the interplay of FC and HT pathways evaluated within a fully consistent treatment of multipolar light–matter coupling and the metal-modified electromagnetic response.

Our results suggest that electronically forbidden resonance TERS offers a practical route to symmetry-sensitive vibrational imaging that is difficult to achieve under electronically allowed resonances. We hope this work will motivate experimental studies of forbidden-resonance TERS and provide a useful theoretical basis for nanoscale vibrational imaging.

## Supplementary Material



## Notes

Detailed theoretical formulation of the TERS Raman-scattering framework; derivation and relation to the Kramers–Heisenberg–Dirac formula; Herzberg–Teller expansion and nonresonant Raman approximation; applicability and limitations of the model; computational details; additional TERS maps and signal-level estimates; and analysis of transition-dipole components, molecular tilt effects, and control calculations on self-consistent electrodynamics (PDF)

## References

[1] Fleischmann M., Hendra P., McQuillan A. (1974). Raman spectra of pyridine adsorbed
at a silver electrode. Chem. Phys. Lett..

[2] Albrecht M. G., Creighton J. A. (1977). Anomalously
intense Raman spectra of pyridine at a
silver electrode. J. Am. Chem. Soc..

[3] Jeanmaire D. L., Van Duyne R. P. (1977). Surface
raman spectroelectrochemistry: Part I. Heterocyclic,
aromatic, and aliphatic amines adsorbed on the anodized silver electrode. Journal of Electroanalytical Chemistry and Interfacial Electrochemistry.

[4] Nie S., Emory S. R. (1997). Probing Single Molecules
and Single Nanoparticles by
Surface-Enhanced Raman Scattering. Science.

[5] Xu H., Bjerneld E. J., Käll M., Börjesson L. (1999). Spectroscopy
of Single Hemoglobin Molecules by Surface Enhanced Raman Scattering. Phys. Rev. Lett..

[6] Vosgröne T., Meixner A. (2004). Surface and resonance enhanced micro-Raman
spectroscopy
of xanthene dyes at the single-molecule level. J. Lumin..

[7] Simoncelli S., Roller E.-M., Urban P., Schreiber R., Turberfield A. J., Liedl T., Lohmüller T. (2016). Quantitative
Single-Molecule Surface-Enhanced Raman Scattering by Optothermal Tuning
of DNA Origami-Assembled Plasmonic Nanoantennas. ACS Nano.

[8] Itoh T., Yamamoto Y. S., Kitahama Y., Balachandran J. (2017). One-dimensional
plasmonic hotspots located between silver nanowire dimers evaluated
by surface-enhanced resonance Raman scattering. Phys. Rev. B.

[9] Benz F., Schmidt M. K., Dreismann A., Chikkaraddy R., Zhang Y., Demetriadou A., Carnegie C., Ohadi H., de Nijs B., Esteban R., Aizpurua J., Baumberg J. J. (2016). Single-molecule
optomechanics in “picocavities”. Science.

[10] Tiwari S., Khandelwal U., Sharma V., Kumar G. P. (2021). Single Molecule
Surface Enhanced Raman Scattering in a Single Gold Nanoparticle-Driven
Thermoplasmonic Tweezer. J. Phys. Chem. Lett..

[11] Wessel J. (1985). Surface-enhanced
optical microscopy. J. Opt. Soc. Am. B.

[12] Stöckle R. M., Suh Y. D., Deckert V., Zenobi R. (2000). Nanoscale chemical
analysis by tip-enhanced Raman spectroscopy. Chem. Phys. Lett..

[13] Hayazawa N., Inouye Y., Sekkat Z., Kawata S. (2000). Metallized
tip amplification
of near-field Raman scattering. Opt. Commun..

[14] Anderson M. S. (2000). Locally
enhanced Raman spectroscopy with an atomic force microscope. Appl. Phys. Lett..

[15] Pettinger B., Picardi G., Schuster R., Ertl G. (2000). Surface Enhanced Raman
Spectroscopy: Towards Single Molecule Spectroscopy. Electrochemistry.

[16] Zhang R., Zhang Y., Dong Z. C., Jiang S., Zhang C., Chen L. G., Zhang L., Liao Y., Aizpurua J., Luo Y., Yang J. L., Hou J. G. (2013). Chemical mapping of a single molecule
by plasmon-enhanced Raman scattering. Nature.

[17] Lee J., Crampton K. T., Tallarida N., Apkarian V. A. (2019). Visualizing vibrational
normal modes of a single molecule with atomically confined light. Nature.

[18] Jaculbia R. B., Imada H., Miwa K., Iwasa T., Takenaka M., Yang B., Kazuma E., Hayazawa N., Taketsugu T., Kim Y. (2020). Single-molecule resonance Raman effect
in a plasmonic nanocavity. Nat. Nanotechnol..

[19] Xu J., Zhu X., Tan S., Zhang Y., Li B., Tian Y., Shan H., Cui X., Zhao A., Dong Z., Yang J., Luo Y., Wang B., Hou J. G. (2021). Determining
structural and chemical heterogeneities of surface species at the
single-bond limit. Science.

[20] Kong F.-F., Tian X.-J., Zhang Y., Yu Y.-J., Jing S.-H., Zhang Y., Tian G.-J., Luo Y., Yang J.-L., Dong Z.-C., Hou J. G. (2021). Probing intramolecular
vibronic coupling
through vibronic-state imaging. Nat. Commun..

[21] de
Campos Ferreira R. C., Sagwal A., Doležal J., Canola S., Merino P., Neuman T., Švec M. (2024). Resonant Tip-Enhanced
Raman Spectroscopy of a Single-Molecule Kondo System. ACS Nano.

[22] Vasilev K., Canola S., Scheurer F., Boeglin A., Lotthammer F., Chérioux F., Neuman T., Schull G. (2024). Exploring the Role
of Excited States’ Degeneracy on Vibronic Coupling with Atomic-Scale
Optics. ACS Nano.

[23] Moskovits M., DiLella D. P., Maynard K. J. (1988). Surface
Raman spectroscopy of a number
of cyclic aromatic molecules adsorbed on silver: selection rules and
molecular reorientation. Langmuir.

[24] Meng L., Yang Z., Chen J., Sun M. (2015). Effect of Electric
Field Gradient on Sub-nanometer Spatial Resolution of Tip-enhanced
Raman Spectroscopy. Sci. Rep..

[25] Wang C.-F., Cheng Z., O’Callahan B.
T., Crampton K. T., Jones M. R., El-Khoury P. Z. (2020). Tip-Enhanced Multipolar Raman Scattering. J. Phys. Chem. Lett..

[26] Takase M., Ajiki H., Mizumoto Y., Komeda K., Nara M., Nabika H., Yasuda S., Ishihara H., Murakoshi K. (2013). Selection-rule
breakdown in plasmon-induced electronic excitation of an isolated
single-walled carbon nanotube. Nat. Photonics.

[27] Albrecht A. C. (1961). On the
Theory of Raman Intensities. J. Chem. Phys..

[28] Duan S., Tian G., Ji Y., Shao J., Dong Z., Luo Y. (2015). Theoretical Modeling
of Plasmon-Enhanced Raman Images of a Single
Molecule with Subnanometer Resolution. J. Am.
Chem. Soc..

[29] Duan S., Tian G., Xie Z., Luo Y. (2017). Gauge invariant theory
for super high resolution Raman images. J. Chem.
Phys..

[30] Xie Z., Duan S., Tian G., Wang C.-K., Luo Y. (2018). Theoretical
modeling of tip-enhanced resonance Raman images of switchable azobenzene
molecules on Au(111). Nanoscale.

[31] Morton S. M., Jensen L. (2010). A discrete interaction
model/quantum mechanical method
for describing response properties of molecules adsorbed on metal
nanoparticles. J. Chem. Phys..

[32] Chulhai D. V., Jensen L. (2013). Determining Molecular
Orientation With Surface-Enhanced
Raman Scattering Using Inhomogenous Electric Fields. J. Phys. Chem. C.

[33] Payton J. L., Morton S. M., Moore J. E., Jensen L. (2014). A Hybrid Atomistic
Electrodynamics–Quantum Mechanical Approach for Simulating
Surface-Enhanced Raman Scattering. Acc. Chem.
Res..

[34] Liu P., Chulhai D. V., Jensen L. (2017). Single-Molecule
Imaging Using Atomistic
Near-Field Tip-Enhanced Raman Spectroscopy. ACS Nano.

[35] Chen X., Liu P., Hu Z., Jensen L. (2019). High-resolution tip-enhanced Raman
scattering probes sub-molecular density changes. Nat. Commun..

[36] Zhang Y., Dong Z.-C., Aizpurua J. (2021). Theoretical
treatment of single-molecule
scanning Raman picoscopy in strongly inhomogeneous near fields. J. Raman Spectrosc..

[37] Takenaka M., Taketsugu T., Iwasa T. (2021). Theoretical method for near-field
Raman spectroscopy with multipolar Hamiltonian and real-time-TDDFT:
Application to on- and off-resonance tip-enhanced Raman spectroscopy. J. Chem. Phys..

[38] Wiser N. (1963). Dielectric
Constant with Local Field Effects Included. Phys. Rev..

[39] Cho, K. Optical response of nanostructures: microscopic nonlocal theory; Springer, 2003; Vol. 139.

[40] Tomoshige Y., Tamura M., Yokoyama T., Ishihara H. (2025). Enhanced photoluminescence
of strongly coupled single molecule-plasmonic nanocavity: analysis
of spectral modifications using nonlocal response theory. Nanophotonics.

[41] Zuev V. S., Frantsesson A. V., Gao J., Eden J. G. (2005). Enhancement
of Raman
scattering for an atom or molecule near a metal nanocylinder: Quantum
theory of spontaneous emission and coupling to surface plasmon modes. J. Chem. Phys..

[42] Gu W., Choi H., Kim K. K. (2007). A Quantum Mechanical Theory for Single
Molecule–Single Nanoparticle Surface Enhanced Raman Scattering. J. Phys. Chem. A.

[43] Dung H. T., Knöll L., Welsch D.-G. (2000). Spontaneous decay in the presence
of dispersing and absorbing bodies: General theory and application
to a spherical cavity. Phys. Rev. A.

[44] Wubs M., Suttorp L. G., Lagendijk A. (2004). Multiple-scattering
approach to interatomic
interactions and superradiance in inhomogeneous dielectrics. Phys. Rev. A.

[45] Yao P., Van Vlack C., Reza A., Patterson M., Dignam M. M., Hughes S. (2009). Ultrahigh Purcell factors and Lamb
shifts in slow-light metamaterial waveguides. Phys. Rev. B.

[46] Kamandar
Dezfouli M., Hughes S. (2017). Quantum Optics Model of Surface-Enhanced
Raman Spectroscopy for Arbitrarily Shaped Plasmonic Resonators. ACS Photonics.

[47] Dirac P. A. M. (1927). The
quantum theory of the emission and absorption of radiation. Proceedings of the Royal Society of London. Series A, Containing
Papers of a Mathematical and Physical Character.

[48] Trautmann S., Aizpurua J., Götz I., Undisz A., Dellith J., Schneidewind H., Rettenmayr M., Deckert V. (2017). A classical description
of subnanometer resolution by atomic features in metallic structures. Nanoscale.

[49] Johnson P. M., Xu H., Sears T. J. (2006). The calculation of vibrational intensities in forbidden
electronic transitions. J. Chem. Phys..

[50] Frisch, M. J. ; Gaussian16, rev. C.01; Gaussian, Inc.: Wallingford, CT, 2016.

[51] Manian A., Shaw R. A., Lyskov I., Wong W., Russo S. P. (2021). Modeling
radiative and non-radiative pathways at both the Franck–Condon
and Herzberg–Teller approximation level. J. Chem. Phys..

[52] Manian A., Russo S. P. (2022). The dominant nature of Herzberg–Teller terms
in the photophysical description of naphthalene compared to anthracene
and tetracene. Sci. Rep..

[53] Cerezo J., Santoro F. (2023). FCclasses3: Vibrationally-resolved
spectra simulated
at the edge of the harmonic approximation. J.
Comput. Chem..

[54] Macak P., Luo Y., Ågren H. (2000). Simulations
of vibronic profiles in two-photon absorption. Chem. Phys. Lett..

[55] Domcke W., Cederbaum L. S. (1977). Theory of the vibrational structure
of resonances in
electron-molecule scattering. Phys. Rev. A.

[56] Goodman J. J., Draine B. T., Flatau P. J. (1991). Application
of fast-Fourier-transform
techniques to the discrete-dipole approximation. Opt. Lett..

