# Supplementary material for: Electronically Forbidden Raman Pathways Create a New Contrast Mechanism in Single-Molecule TERS
Source: Nano Lett. 2026 Mar 16;26(14):4613–20. doi: 10.1021/acs.nanolett.5c06490 (PMC13088357; doi:10.1021/acs.nanolett.5c06490)
Supplement: Supplementary file 1 [file nl5c06490_si_001.pdf]

# Supporting Information of "Electronically Forbidden Raman Pathways Create a New Contrast Mechanism in Single-Molecule TERS"

Hiroyuki Ikagawa,[†] Mamoru Tamura,[*,‡,¶,†,§] and Hajime Ishihara[*,§,‖,⊥,†]

†*Department of Materials Engineering Science, The University of Osaka, 1-3 Machikaneyama-cho, Toyonaka, Osaka 560-8531, Japan*

‡*Department of Physics and Astronomy, School of Science, Kwansei Gakuin University, 1 Gakuen Uegahara, Sanda, Hyogo 669-1330, Japan*

¶*Research Institute for Light-induced Acceleration System (RILACS), Osaka Metropolitan University, 1-2 Gakuencho, Nakaku, Sakai, Osaka 599-8570, Japan*

§*Research Organization of Science and Technology, Ritsumeikan University, 1-1-1 Nojihigashi, Kusatsu, Shiga 525-8577, Japan*

‖*SANKEN, The University of Osaka, 8-1, Mihogaoka, Ibaraki, Osaka 567-0047, Japan*

⊥*Ritsumeikan Semiconductor Application research center (RISA), Ritsumeikan University, 1-1-1 Nojihigashi, Kusatsu, Shiga 525-8577, Japan*

E-mail: mamoru.tamura@kwansei.ac.jp; ishi@mp.es.osaka-u.ac.jp

# 1 Theoretical formulation

## 1.1 Context and provenance

The derivation in Sections 1.2–1.5 follows the standard perturbative backbone of Raman theory, i.e., the Kramers–Heisenberg–Dirac (KHD) dispersion formula and its vibronic/Herzberg–Teller (Albrecht-type) expansion.[1,2] To obtain submolecular picocavity-TERS maps, however, one must treat (i) the spatially structured (nonlocal) molecular polarization and (ii) the full junction electrodynamics with self-consistent backaction on the same footing; treating either ingredient in isolation is insufficient to connect the vibronic Raman pathway to a measurable, position-dependent Stokes signal. Accordingly, we combine established transition-dipole-density/nonlocal and Green-tensor-based formalisms into a single, internally consistent Raman-scattering expression, applied consistently at both the incident and Stokes frequencies, that yields directly the Stokes photon flux and maps.[3–5] Sections 1.2–1.5 detail these ingredients and their combination, leading to the final expressions used for the Stokes photon flux and maps. To make the role of this unified treatment transparent, we also include diagnostic calculations in Sec. 7 where individual ingredients are selectively simplified, showing that the key contrast mechanism discussed in this work is captured only when the full formulation is retained.

## 1.2 Molecular nonlocal polarization

Here we show the Hamiltonian used by our TERS theory and describe the derivation of the nonlocal polarization.

The unperturbed Hamiltonian $\hat{H}_0$ is described as

$$\hat{H}_0 = \hat{H}_{\mathrm{mol}} + \hat{H}_{\mathrm{rad}} \tag{S1}$$

$$\hat{H}_{\mathrm{mol}} = \sum_{n} \hbar\omega_n \hat{\sigma}_{nn}, \tag{S2}$$

$$\hat{H}_{\mathrm{rad}} = \sum_{\eta} \hbar\Omega_\eta \hat{a}^\dagger_\eta \hat{a}_\eta, \tag{S3}$$

where $\hat{H}_{\mathrm{mol}}$ and $\hat{H}_{\mathrm{rad}}$ describe the Hamiltonian of a sample molecule and photons, respectively. The ladder operator $\hat{\sigma}_{nm}$ is described as

$$\hat{\sigma}_{nm} = |n\rangle \langle m| \tag{S4}$$

where $|n\rangle$ is a state vector for molecular $n$ state. Within the Born–Oppenheimer approximation,[6] these states can be written as direct products of an electronic state $|S_n\rangle$ and a vibrational state $|v_{S_n}\rangle$, i.e., $|n\rangle = |S_n\rangle |v_{S_n}\rangle$. $\hat{a}^\dagger_\eta$ and $\hat{a}_\eta$ are creation and annihilation operator of $\eta$ mode photons, respectively. These operators satisfy bosonic commutation relation, following as

$$[\hat{a}_\eta, \hat{a}_{\eta'}] = 0, \quad [\hat{a}^\dagger_\eta, \hat{a}^\dagger_{\eta'}] = 0, \quad [\hat{a}_\eta, \hat{a}^\dagger_{\eta'}] = \delta_{\eta,\eta'}. \tag{S5}$$

$\hat{V}$ represents the interaction between the molecule and photons, described as

$$\hat{V} = -\int d\boldsymbol{r} \hat{\boldsymbol{P}}(\boldsymbol{r}) \cdot \hat{\boldsymbol{E}}(\boldsymbol{r}) \tag{S6}$$

where $\hat{\boldsymbol{P}}(\boldsymbol{r})$ is a molecular polarization operator and $\hat{\boldsymbol{E}}(\boldsymbol{r})$ is an electric field operator. $\hat{\boldsymbol{P}}(\boldsymbol{r})$ is described as

$$\hat{\boldsymbol{P}}(\boldsymbol{r}) = \sum_{n,m} \boldsymbol{\mathcal{P}}_{nm}(\boldsymbol{r}) \hat{\sigma}_{nm}, \tag{S7}$$

where $\boldsymbol{\mathcal{P}}_{nm}(\boldsymbol{r})$ is density of molecular transition dipole moment between $n$ and $m$ molecular

states. The electric field operator can be written as an expansion over the modes $\eta$ in terms of the corresponding mode operators and eigenfunctions $\boldsymbol{f}_\eta(\boldsymbol{r})$,[5,7] as follows:

$$\hat{\boldsymbol{E}}(\boldsymbol{r}) = \sum_\eta \alpha_\eta i \boldsymbol{f}_\eta(\boldsymbol{r}) \hat{a}_\eta + \text{H. C.}, \quad \alpha_\eta = \sqrt{\frac{\hbar\Omega_\eta}{2\varepsilon_0}}. \tag{S8}$$

$\boldsymbol{f}_\eta(\boldsymbol{r})$ satisfies following Maxwell's equation,

$$\boldsymbol{\nabla} \times \boldsymbol{\nabla} \times \boldsymbol{f}_\eta(\boldsymbol{r}) - \varepsilon_{\text{metal}}(\boldsymbol{r}, \Omega_\eta) \frac{\Omega_\eta^2}{c^2} \boldsymbol{f}_\eta(\boldsymbol{r}) = \boldsymbol{0}. \tag{S9}$$

where $\varepsilon_{\text{metal}}(\boldsymbol{r}, \omega)$ denotes the dielectric function of the metallic structure. To treat expectation values of operators, we introduce the density operator $\hat{\rho}(t)$ and consider the following quantum master equation. The quantum master equation with Born-Markov approximation is described as[7]

$$\frac{d}{dt}\hat{\rho}(t) = \frac{1}{i\hbar}[\hat{H}_0 + \hat{V}, \hat{\rho}(t)] + \mathcal{L}\hat{\rho}(t). \tag{S10}$$

The second term of R. H. S. in Eq. (S10) represents relaxation, described as

$$\mathcal{L}\hat{\rho}(t) = \sum_{n,m} \frac{\gamma_{nm}^{\text{damp}}}{2} \Big[ 2\hat{\sigma}_{nm}\hat{\rho}(t)\hat{\sigma}_{nm}^\dagger - \big\{ \hat{\sigma}_{nm}^\dagger \hat{\sigma}_{nm}, \hat{\rho}(t) \big\} \Big] - \sum_{n,m} \frac{\gamma_{nm}^{\text{phase}}}{2} \Big[ \hat{\sigma}_{nm}^\dagger \hat{\sigma}_{nm}, \big[ \hat{\sigma}_{nm}^\dagger \hat{\sigma}_{nm}, \hat{\rho}(t) \big] \Big], \tag{S11}$$

where $\gamma_{nm}^{\text{damp}}$ and $\gamma_{nm}^{\text{phase}}$ are damping and pure dephasing rate, respectively.

We treat $\hat{V}$ as a perturbation and consider the time evolution of the expectation value of the ladder operator $\hat{\sigma}_{nm}$ to first order in $\hat{V}$. The quantum master equation of this operator

is described as

$$\frac{d}{dt}\langle\hat{\sigma}_{nm}(t)\rangle = \mathrm{Tr}\left[\frac{d}{dt}\hat{\rho}(t)\hat{\sigma}_{nm}\right]$$
$$= \mathrm{Tr}\left[\frac{1}{i\hbar}\hat{\rho}(t)\left[\hat{\sigma}_{nm},\hat{H}_0\right] + \mathcal{L}\hat{\rho}(t)\hat{\sigma}_{nm}\right]$$
$$= -(i\omega_{mn}+\Gamma_{nm})\langle\hat{\sigma}_{nm}(t)\rangle + \frac{i}{\hbar}\big(\langle\hat{\sigma}_{nn}(0)\rangle - \langle\hat{\sigma}_{mm}(0)\rangle\big)\int d\boldsymbol{r}\boldsymbol{\mathcal{P}}_{mn}(\boldsymbol{r})\cdot\langle\hat{\boldsymbol{E}}(\boldsymbol{r},t)\rangle\,, \tag{S12}$$

where $\Gamma_{nm} = \sum_{n'}\{\gamma^{\text{damp}}_{n'm} + \gamma^{\text{damp}}_{n'n} + \gamma^{\text{phase}}_{n'm} + \gamma^{\text{phase}}_{n'n}\}/2$ is total relaxation rate, and $\langle\hat{\sigma}_{nn}(0)\rangle$ denotes the population of state $n$ in the initial state before the perturbation is applied. For the second term on the right-hand side of Eq. (S12), we used the following approximation.

$$\langle\hat{\sigma}_{nm}(t)\hat{\boldsymbol{E}}(\boldsymbol{r},t)\rangle \simeq \delta_{nm}\langle\hat{\sigma}_{nn}(0)\rangle\,\langle\hat{\boldsymbol{E}}(\boldsymbol{r},t)\rangle\,. \tag{S13}$$

This approximation is based on the fact that the incident light is a laser field in a coherent state, so that the field at the incident frequency can be treated classically. Moreover, within the linear-response regime, the ladder operator appearing in this field term can be replaced by its initial-state value, which is independent of the field. In particular, the off-diagonal elements are assumed to be negligible and are therefore omitted.

Denoting the incident frequency by $\omega_i$, the expectation value of the electric field and polarization operator can be written as follows:

$$\langle\hat{\boldsymbol{E}}(\boldsymbol{r},t)\rangle = e^{-i\omega_i t}\boldsymbol{E}(\boldsymbol{r},\omega_i) + \text{C. C.}, \tag{S14}$$

$$\langle\hat{\boldsymbol{P}}(\boldsymbol{r},t)\rangle = e^{-i\omega_i t}\boldsymbol{P}(\boldsymbol{r},\omega_i) + \text{C. C.}. \tag{S15}$$

By evaluating $\boldsymbol{P}(\boldsymbol{r},\omega_i)$ from Eq. (S12), we obtain the following expression:

$$\boldsymbol{P}(\boldsymbol{r},\omega_i) = \varepsilon_0 \int d\boldsymbol{r}' \boldsymbol{\chi}(\boldsymbol{r},\boldsymbol{r}',\omega_i)\boldsymbol{E}(\boldsymbol{r}',\omega_i), \tag{S16}$$

$$\boldsymbol{\chi}(\boldsymbol{r},\boldsymbol{r}',\omega) = \frac{1}{\varepsilon_0}\sum_{n,m}\big(\langle\hat{\sigma}_{nn}(0)\rangle - \langle\hat{\sigma}_{mm}(0)\rangle\big) g_{mn}(\omega)\boldsymbol{\mathcal{P}}_{nm}(\boldsymbol{r})\boldsymbol{\mathcal{P}}_{mn}(\boldsymbol{r}'), \tag{S17}$$

$$g_{mn}(\omega) = \hbar^{-1}(\omega_m - \omega_n - \omega - i\Gamma_{nm})^{-1}. \tag{S18}$$

Furthermore, the electric field is obtained as follows.

$$\boldsymbol{E}(\boldsymbol{r},\omega_i) = \boldsymbol{E}_{\mathrm{inc}}(\boldsymbol{r},\omega_i) + \int d\boldsymbol{r}' \boldsymbol{G}_{\mathrm{vac}}(\boldsymbol{r},\boldsymbol{r}',\omega_i)\{\boldsymbol{P}(\boldsymbol{r}',\omega_i) + \boldsymbol{P}_{\mathrm{metal}}(\boldsymbol{r}',\omega_i)\}, \tag{S19}$$

where $\boldsymbol{G}_{\mathrm{vac}}(\boldsymbol{r},\boldsymbol{r}',\omega)$ is Green's function of Maxwell's equation in vacuum. $\boldsymbol{E}_{\mathrm{inc}}(\boldsymbol{r},\omega_i)$ represent electric field of incident laser. $\boldsymbol{P}_{\mathrm{metal}}(\boldsymbol{r},\omega_i)$ is polarization on metal nanostructures, described as follows:

$$\boldsymbol{P}_{\mathrm{metal}}(\boldsymbol{r},\omega_i) = \varepsilon_0(\varepsilon_{\mathrm{metal}}(\boldsymbol{r},\omega_i) - 1)\boldsymbol{E}(\boldsymbol{r},\omega_i). \tag{S20}$$

By determining the electric field and polarization in Eq. (S16)(S19)(S20) self-consistently, one obtains the local near field $\boldsymbol{E}_{\mathrm{loc}}(\boldsymbol{r},\omega_i)$ as the solution. In this study, we employed the discrete dipole approximation (DDA) to calculate $\boldsymbol{E}_{\mathrm{loc}}(\boldsymbol{r},\omega_i)$.[8]

## 1.3 Signal intensity in TERS

Here, starting from the Hamiltonian and quantum master equation in Eq. (S1)–(S11), we describe the procedure used to derive the expression for the TERS scattering intensity. Within this framework, it is, in principle, possible to estimate the intensity of incoherent

radiation, such as luminescence and Raman scattering. The present theory is based on a Hamiltonian that includes infinitely many photonic modes (Eq. S3); however, by employing a scheme that consolidates these modes into a single Green's function,[5,7] we can describe metal nanostructures of arbitrary shape and multipolar transitions while keeping the computational cost manageable. Moreover, by including both the Franck–Condon (FC) and Herzberg–Teller (HT) transition dipole densities in $\boldsymbol{\mathcal{P}}_{nm}(\boldsymbol{r})$ of Eq. (S7) (see Sec. 1.5 for details) and treating them consistently,[9] we describe resonant TERS while incorporating all effects corresponding to Albrecht ' s theory of resonant Raman scattering.[1]

Below, we outline the perturbative derivation. In our previous work, we evaluated the emission intensity from many emitters by calculating the time rate of change of the number of photons emitted by the radiation (i.e., the photon flux).[10] Because Raman scattering, like luminescence, is an incoherent radiative process, this framework provides a powerful means of evaluation. Accordingly, we performed a perturbative calculation for the photon flux. The photon flux can be decomposed into frequency components as follows.

$$\begin{aligned}\sum_{\eta} \frac{d}{dt} \langle \hat{a}^{\dagger}_{\eta}(t)\hat{a}_{\eta}(t)\rangle &= \sum_{\omega_{s'}} \sum_{\eta\in\omega_{s'}} \frac{d}{dt} \langle \hat{a}^{\dagger}_{\eta}(t)\hat{a}_{\eta}(t)\rangle \\ &= \int_{-\infty}^{\infty} d\omega_s \sum_{\omega_{s'}} \delta(\omega_s - \omega_{s'}) \sum_{\eta\in\omega_{s'}} \frac{d}{dt} \langle \hat{a}^{\dagger}_{\eta}(t)\hat{a}_{\eta}(t)\rangle \\ &= \int_{0}^{\infty} d\omega_s \sum_{\omega_{s'}} \{\delta(\omega_s - \omega_{s'}) + \delta(\omega_s + \omega_{s'})\} \sum_{\eta\in\omega_{s'}} \frac{d}{dt} \langle \hat{a}^{\dagger}_{\eta}(t)\hat{a}_{\eta}(t)\rangle \\ &= \int_{0}^{\infty} d\omega_s S(\omega_s), \end{aligned} \tag{S21}$$

$$S(\omega) = \sum_{\omega_s} \{\delta(\omega - \omega_s) + \delta(\omega + \omega_s)\} \sum_{\eta\in\omega_{s'}} \frac{d}{dt} \langle \hat{a}^{\dagger}_{\eta}(t)\hat{a}_{\eta}(t)\rangle \,, \tag{S22}$$

where the summation symbol $\sum_{\eta\in\omega_{s'}}$ indicates that the sum is taken over all modes $\eta$ whose frequency satisfies $\Omega_{\eta} = \omega_{s'}$. From $S(\omega_s)$, the scattering intensity at the scattered frequency $\omega_s$ can be obtained.

Raman scattering is generally a two-photon process in which the molecule interacts with one incident photon and one scattered photon, and the Raman polarizability is obtained from second-order perturbation theory.[11,12] Accordingly, in the perturbative evaluation of $S(\omega_s)$ (which is proportional to the scattering intensity at frequency $\omega_s$), we compute terms up to fourth order.

The time evolution of the photon number operator is derived from the quantum master equation as follows.

$$\frac{d}{dt}\left\langle \hat{a}^{\dagger}_{\eta}(t)\hat{a}_{\eta}(t)\right\rangle = \sum_{n,m}\frac{\alpha_{\eta}}{\hbar}\left\langle \hat{\sigma}_{nm}(t)\hat{a}_{\eta}(t)\right\rangle \int d\boldsymbol{r}\boldsymbol{\mathcal{P}}_{nm}(\boldsymbol{r})\cdot\boldsymbol{f}_{\eta}(\boldsymbol{r}) + \text{C. C.}. \tag{S23}$$

Furthermore, the expectation value of the two-body operator composed of the ladder and annihilation operators in Eq. (S23) evolves in time as follows.

$$\begin{aligned}
&\frac{d}{dt}\left\langle \hat{\sigma}_{nm}(t)\hat{a}_{\eta}(t)\right\rangle \\
&= \left\{-i(\omega_m - \omega_n + \Omega_{\eta}) - \Gamma_{nm}\right\}\left\langle \hat{\sigma}_{nm}(t)\hat{a}_{\eta}(t)\right\rangle + \frac{\alpha_{\eta}}{\hbar}\sum_{n'}\int d\boldsymbol{r}\boldsymbol{\mathcal{P}}_{mn'}(\boldsymbol{r})\cdot\boldsymbol{f}^{*}_{\eta}(\boldsymbol{r})\left\langle \hat{\sigma}_{nn'}(t)\right\rangle \\
&+ \frac{i}{\hbar}\sum_{n'}\int d\boldsymbol{r}\Big\{\boldsymbol{\mathcal{P}}_{mn'}(\boldsymbol{r})\cdot\left\langle \hat{\sigma}_{nn'}(t)\hat{\boldsymbol{E}}(\boldsymbol{r},t)\hat{a}_{\eta}(t)\right\rangle - \boldsymbol{\mathcal{P}}_{n'n}(\boldsymbol{r})\cdot\left\langle \hat{\sigma}_{n'm}(t)\hat{\boldsymbol{E}}(\boldsymbol{r},t)\hat{a}_{\eta}(t)\right\rangle\Big\}.
\end{aligned} \tag{S24}$$

The third and fourth term on the R. H. S. of Eq. (S24) includes multiphoton processes such as two-photon absorption. The second term on the R. H. S. corresponds to a one-photon process, such as absorption or fluorescence. These terms can be neglected in the present discussion of Raman scattering. Then we obtained following equation:

$$\begin{aligned}
&\frac{d}{dt}\left\langle \hat{\sigma}_{nm}(t)\hat{a}_{\eta}(t)\right\rangle \\
&= \left\{-i(\omega_m - \omega_n + \Omega_{\eta}) - \Gamma_{nm}\right\}\left\langle \hat{\sigma}_{nm}(t)\hat{a}_{\eta}(t)\right\rangle \\
&+ \frac{1}{\hbar}\sum_{n',\eta'}\int d\boldsymbol{r}\alpha_{\eta'}\boldsymbol{f}^{*}_{\eta'}(\boldsymbol{r})\cdot\Big\{\boldsymbol{\mathcal{P}}_{mn'}(\boldsymbol{r})\ \left\langle \hat{\sigma}_{nn'}(t)\hat{a}^{\dagger}_{\eta'}(t)\hat{a}_{\eta}(t)\right\rangle - \boldsymbol{\mathcal{P}}_{n'n}(\boldsymbol{r})\left\langle \hat{\sigma}_{n'm}(t)\hat{a}^{\dagger}_{\eta'}(t)\hat{a}_{\eta}(t)\right\rangle\Big\},
\end{aligned} \tag{S25}$$

The time evolution of the expectation value of the three-body operator involving the ladder, creation, and annihilation operators in Eq. (S25) is given as follows.

$$
\begin{aligned}
&\frac{d}{dt}\left\langle \hat{\sigma}_{nm}(t)\hat{a}^{\dagger}_{\eta'}(t)\hat{a}_{\eta}(t)\right\rangle \\
&= \{-i(\omega_m-\omega_n-\Omega_{\eta'}+\Omega_{\eta})-\Gamma_{nm}\}\left\langle \hat{\sigma}_{nm}(t)\hat{a}^{\dagger}_{\eta'}(t)\hat{a}_{\eta}(t)\right\rangle \\
&+\frac{1}{\hbar}\sum_{n'}\int d\boldsymbol{r}\boldsymbol{\mathcal{P}}_{mn'}(\boldsymbol{r})\cdot\Big\{\alpha_{\eta}\boldsymbol{f}^{*}_{\eta}(\boldsymbol{r})\left\langle \hat{\sigma}_{nn'}(t)\hat{a}^{\dagger}_{\eta'}(t)\right\rangle+\alpha_{\eta'}\boldsymbol{f}_{\eta'}(\boldsymbol{r})\left\langle \hat{\sigma}_{nn'}(t)\hat{a}_{\eta}(t)\right\rangle\Big\} \\
&+\frac{1}{\hbar}\sum_{n',\eta''}\int d\boldsymbol{r}\alpha_{\eta''}\Big\{ \\
&\quad\boldsymbol{\mathcal{P}}_{mn'}(\boldsymbol{r})\cdot\boldsymbol{f}^{*}_{\eta''}(\boldsymbol{r})\left\langle \hat{\sigma}_{nn'}(t)\hat{a}^{\dagger}_{\eta''}(t)\hat{a}^{\dagger}_{\eta'}(t)\hat{a}_{\eta}(t)\right\rangle-\boldsymbol{\mathcal{P}}_{n'n}(\boldsymbol{r})\cdot\boldsymbol{f}^{*}_{\eta''}(\boldsymbol{r})\left\langle \hat{\sigma}_{n'm}(t)\hat{a}^{\dagger}_{\eta''}(t)\hat{a}^{\dagger}_{\eta'}(t)\hat{a}_{\eta}(t)\right\rangle \\
&-\boldsymbol{\mathcal{P}}_{mn'}(\boldsymbol{r})\cdot\boldsymbol{f}_{\eta''}(\boldsymbol{r})\left\langle \hat{\sigma}_{nn'}(t)\hat{a}_{\eta''}(t)\hat{a}^{\dagger}_{\eta'}(t)\hat{a}_{\eta}(t)\right\rangle+\boldsymbol{\mathcal{P}}_{n'n}(\boldsymbol{r})\cdot\boldsymbol{f}_{\eta''}(\boldsymbol{r})\left\langle \hat{\sigma}_{n'm}(t)\hat{a}_{\eta''}(t)\hat{a}^{\dagger}_{\eta'}(t)\hat{a}_{\eta}(t)\right\rangle \\
&\Big\}. \qquad \text{(S26)}
\end{aligned}
$$

Eq. (S26) contains the operator of $\eta'$ and $\eta''$ mode photons. We assumed that the modes involved in scattering into different modes of $\eta$ correspond to modes at the incident frequency $\omega_i$. In the previous section, we assumed that this mode can be treated classically. We make the same assumption here and suppose that the following approximation holds.

$$
\begin{aligned}
&\left\langle \hat{\sigma}_{nm}(t)\hat{a}^{\dagger}_{\eta'}(t)\right\rangle \simeq \left\langle \hat{\sigma}_{nm}(t)\right\rangle \left\langle \hat{a}^{\dagger}_{\eta'}(t)\right\rangle \\
&\simeq \left\langle \hat{a}^{\dagger}_{\eta'}(t)\right\rangle \left( \left\langle \hat{\sigma}_{nn}(0)\right\rangle - \left\langle \hat{\sigma}_{mm}(0)\right\rangle\right) \\
&\times \int d\boldsymbol{r}\boldsymbol{\mathcal{P}}_{mn}(\boldsymbol{r})\cdot\{g_{mn}(\omega_i)\boldsymbol{E}_{\text{loc}}(\boldsymbol{r},\omega_i)e^{-i\omega_i t}+g_{mn}(-\omega_i)\boldsymbol{E}_{\text{loc}}(\boldsymbol{r},-\omega_i)e^{i\omega_i t}\}. \qquad \text{(S27)}
\end{aligned}
$$

where $\boldsymbol{E}_{\text{loc}}(\boldsymbol{r},\omega_i)$ is the near field obtained as the solution of Eq. (S16)(S19)(S20). Furthermore, the terms involving $\boldsymbol{E}_{\text{loc}}(\boldsymbol{r},\omega_i)e^{i\omega_i t}$ in Eq. (S27) can be neglected under the rotating-wave approximation, since $\langle \hat{a}^{\dagger}_{\eta'}(t)\rangle$ oscillates at the same angular frequency. Furthermore, the third term including $\langle \hat{\sigma}_{nn'}(t)\hat{a}_{\eta}(t)\rangle$ on the R. H. S. of Eq. (S26) contributes to stimulated emission. Therefore, we neglect these terms. Moreover, in accordance with the fact that the Raman scattering intensity is proportional to the squared modulus of the incident

electric field, we retain, among the fourth to seventh terms, only those containing the annihilation operator of mode $\eta''$ and the creation operator of mode $\eta'$. By applying the above approximations to Eq. (S26), we obtain the following expression.

$$
\begin{aligned}
&\frac{d}{dt}\langle\hat{\sigma}_{nm}(t)\hat{a}^{\dagger}_{\eta'}(t)\hat{a}_{\eta}(t)\rangle \\
&\simeq \{-i(\omega_m-\omega_n-\omega_i+\Omega_\eta)-\Gamma_{nm}\}\langle\hat{\sigma}_{nm}(t)\hat{a}^{\dagger}_{\eta'}(t)\hat{a}_{\eta}(t)\rangle \\
&+\frac{1}{\hbar}\sum_{n'}\int d\boldsymbol{r}\boldsymbol{\mathcal{P}}_{mn'}(\boldsymbol{r})\cdot\Big\{\alpha_\eta\boldsymbol{f}^{*}_{\eta}(\boldsymbol{r})\langle\hat{a}^{\dagger}_{\eta'}(t)\rangle\left(\langle\hat{\sigma}_{nn}(0)\rangle-\langle\hat{\sigma}_{n'n'}(0)\rangle\right) \\
&\qquad\qquad\times\Big(\boldsymbol{\mathcal{P}}_{n'n}(\boldsymbol{r})\cdot\{g_{n'n}(\omega_i)e^{-i\omega_i t}\tilde{\boldsymbol{E}}(\boldsymbol{r},\omega_i)\}\Big)\Big\} \\
&-\frac{1}{\hbar}\sum_{n',\eta''}\int d\boldsymbol{r}\alpha_{\eta''}\Big\{\boldsymbol{\mathcal{P}}_{mn'}(\boldsymbol{r})\cdot\boldsymbol{f}_{\eta''}(\boldsymbol{r})\langle\hat{\sigma}_{nn'}(t)\hat{a}_{\eta''}(t)\hat{a}^{\dagger}_{\eta'}(t)\hat{a}_{\eta}(t)\rangle \\
&\qquad\qquad-\boldsymbol{\mathcal{P}}_{n'n}(\boldsymbol{r})\cdot\boldsymbol{f}_{\eta''}(\boldsymbol{r})\langle\hat{\sigma}_{n'm}(t)\hat{a}_{\eta''}(t)\hat{a}^{\dagger}_{\eta'}(t)\hat{a}_{\eta}(t)\rangle\Big\}.
\end{aligned}
\tag{S28}
$$

We also take into account the four-body operator term appearing in Eq. (S28). In evaluating its time evolution, we neglect all terms involving five or more operators. Furthermore, by retaining only the contributions that depend on the creation operator of mode $\eta'$ and the annihilation operator of mode $\eta''$, we obtain the following equation of motion:

$$
\begin{aligned}
\frac{d}{dt}\langle\hat{\sigma}_{nm}(t)\hat{a}_{\eta''}(t)\hat{a}^{\dagger}_{\eta'}(t)\hat{a}_{\eta}(t)\rangle &\simeq \{-i(\omega_m-\omega_n+\Omega_\eta)-\Gamma_{nm}\}\langle\hat{\sigma}_{nm}(t)\hat{a}_{\eta''}(t)\hat{a}^{\dagger}_{\eta'}(t)\hat{a}_{\eta}(t)\rangle \\
&+\frac{\alpha_\eta}{\hbar}\sum_{n'}\langle\hat{\sigma}_{nn}(0)\rangle\langle\hat{a}_{\eta''}(t)\rangle\langle\hat{a}^{\dagger}_{\eta'}(t)\rangle\int d\boldsymbol{r}\boldsymbol{\mathcal{P}}_{mn'}(\boldsymbol{r})\cdot\boldsymbol{f}^{*}_{\eta}(\boldsymbol{r}).
\end{aligned}
\tag{S29}
$$

We sequentially solved Eqs. (S29), (S28) and (S25) under the steady-state approximation. Moreover, for the $\eta'$ modes that can be treated classically, The operators can be rewritten in terms of the local near field $\boldsymbol{E}_{\mathrm{loc}}(\boldsymbol{r},\pm\omega_i)$ obtained as the solution of the coupled equations

in Eq. (S16)(S19)(S20), as follows.

$$i\alpha_{\eta'}\boldsymbol{f}_{\eta'}(\boldsymbol{r})\langle\hat{a}_{\eta'}(t)\rangle = e^{-i\omega_i t}\boldsymbol{E}_{\rm loc}(\boldsymbol{r},\omega_i),\ \ -i\alpha_{\eta'}\boldsymbol{f}^*_{\eta'}(\boldsymbol{r})\langle\hat{a}^\dagger_{\eta'}(t)\rangle = e^{i\omega_i t}\boldsymbol{E}_{\rm loc}(\boldsymbol{r},-\omega_i). \tag{S30}$$

Furthermore, using the eigenfunctions $\boldsymbol{f}_\eta(\boldsymbol{r})$ of the scattering mode $\eta$, we can introduce the renormalized Green's function as follows. [5,7]

$$\boldsymbol{G}_{\rm ren}(\boldsymbol{r},\boldsymbol{r}',\omega) = \sum_\eta \frac{i\pi\Omega_\eta \boldsymbol{f}_\eta(\boldsymbol{r})\boldsymbol{f}^*_\eta(\boldsymbol{r}')}{\varepsilon_0}\{\delta(\omega-\Omega_\eta)+\delta(\omega+\Omega_\eta)\}. \tag{S31}$$

$\boldsymbol{G}_{\rm ren}(\boldsymbol{r},\boldsymbol{r}',\omega)$ satisfies

$$\{(\boldsymbol{\nabla}\times\boldsymbol{\nabla}\times) - \varepsilon_{\rm metal}(\boldsymbol{r},\omega)\omega^2/c^2\}\boldsymbol{G}_{\rm ren}(\boldsymbol{r},\boldsymbol{r}',\omega) = -\mu_0\omega^2\delta(\boldsymbol{r}-\boldsymbol{r}'). \tag{S32}$$

From the above procedure, the expression for $S(\omega_s)$ is given by

$$\begin{aligned} S(\omega_s) = \frac{1}{\pi}\sum_{n,m,n',m'} \mathrm{Re}\big[ & \\ & g_{mn}(-\omega_s)g_{m'n}(\omega_i)g^*_{nn'}(-\omega_i+\omega_s)(\langle\hat{\sigma}_{nn}(0)\rangle - \langle\hat{\sigma}_{m'm'}(0)\rangle)X_{nm,n'm'}(\omega_s)F_{mn',m'n}(\omega_i) \\ & + g_{mn}(-\omega_s)g_{m'n}(-\omega_s)g^*_{nn'}(-\omega_i+\omega_s)\langle\hat{\sigma}_{nn}(0)\rangle X_{nm,m'n}(\omega_s)F_{mn',n'm'}(\omega_i) \\ & + g_{mn}(-\omega_s)g_{n'm'}(-\omega_s)g_{n'n}(\omega_i-\omega_s)\langle\hat{\sigma}_{m'm'}(0)\rangle X_{nm,n'm'}(\omega_s)F_{mn',m'n}(\omega_i) \\ & + g_{mn}(-\omega_s)g_{m'n'}(\omega_i)g_{mn'}(\omega_i-\omega_s)(\langle\hat{\sigma}_{n'n'}(0)\rangle - \langle\hat{\sigma}_{m'm'}(0)\rangle)X_{nm,mm'}(\omega)F_{n'n,m'n'}(\omega_i) \\ & + g_{mn}(-\omega_s)g_{m'n'}(-\omega_s)g_{mn'}(\omega_i-\omega_s)\langle\hat{\sigma}_{n'n'}(0)\rangle X_{nm,m'n'}(\omega_s)F_{n'n,mm'}(\omega_i) \\ & + g_{mn}(-\omega_s)g_{mm'}(-\omega_s)g^*_{mn'}(-\omega_i+\omega_s)\langle\hat{\sigma}_{m'm'}(0)\rangle X_{nm,mm'}(\omega_s)F_{n'n,m'n'}(\omega_i) \\ \big], & \end{aligned} \tag{S33}$$

$$X_{nm,n'm'}(\omega) = \iint d\boldsymbol{r}_1 d\boldsymbol{r}_2 \boldsymbol{\mathcal{P}}_{nm}(\boldsymbol{r}_1)\boldsymbol{G}_{\rm ren}(\boldsymbol{r}_1,\boldsymbol{r}_2,\omega)\boldsymbol{\mathcal{P}}_{n'm'}(\boldsymbol{r}_2), \tag{S34}$$

$$F_{nm,n'm'}(\omega_i) = \left(\int d\boldsymbol{r}_1 \boldsymbol{\mathcal{P}}_{nm}(\boldsymbol{r}_1) \cdot \boldsymbol{E}_{\mathrm{loc}}(\boldsymbol{r}_1, -\omega_i)\right)\left(\int d\boldsymbol{r}_2 \boldsymbol{\mathcal{P}}_{n'm'}(\boldsymbol{r}_2) \cdot \boldsymbol{E}_{\mathrm{loc}}(\boldsymbol{r}_2, \omega_i)\right). \tag{S35}$$

Note that the Green's function in Eq. (S31) is not the retarded Green's function itself. However, by treating $\boldsymbol{\mathcal{P}}_{nm}(\boldsymbol{r})$ as a real function, $X_{nm,n'm'}(\omega)$ can be rewritten in terms of the retarded Green's function $\boldsymbol{G}^{\mathrm{ret}}_{\mathrm{ren}}(\boldsymbol{r}, \boldsymbol{r}', \omega)$ as follows.

$$X_{nm,n'm'}(\omega) = 2\mathrm{Im}\left[\iint d\boldsymbol{r} d\boldsymbol{r}' \boldsymbol{\mathcal{P}}_{nm}(\boldsymbol{r}) \boldsymbol{G}^{\mathrm{ret}}_{\mathrm{ren}}(\boldsymbol{r}, \boldsymbol{r}', \omega) \boldsymbol{\mathcal{P}}_{n'm'}(\boldsymbol{r}')\right] \tag{S36}$$

Retaining in Eq. (S33) only the contribution that attains a local maximum under the condition for Stokes Raman scattering, $(\omega_2 - \omega_1) - (\omega_i - \omega_s) \to 0$, where $n = 1$ and $n = 2$ denotes the ground and vibrational excited states,respectively), the scattering intensity is given by:

$$\begin{aligned} S(\omega_s) = \frac{1}{\pi} \sum_{n,m} \mathrm{Re}\big[ \\ & g_{m1}(-\omega_s) g_{n1}(\omega_i) g^*_{12}(-\omega_i + \omega_s) (\langle \hat{\sigma}_{11}(0) \rangle - \langle \hat{\sigma}_{nn}(0) \rangle) X_{1m,2n}(\omega_s) F_{m2,n1}(\omega_i) \\ & + g_{m1}(-\omega_s) g_{n1}(-\omega_s) g^*_{12}(-\omega_i + \omega_s) \langle \hat{\sigma}_{11}(0) \rangle X_{1m,n1}(\omega_s) F_{m2,2n}(\omega_i) \\ & + g_{m1}(-\omega_s) g_{2n}(-\omega_s) g_{21}(\omega_i - \omega_s) \langle \hat{\sigma}_{nn}(0) \rangle X_{1m,2n}(\omega_s) F_{m2,n1}(\omega_i) \\ & + g_{2n}(-\omega_s) g_{m1}(\omega_i) g_{21}(\omega_i - \omega_s) (\langle \hat{\sigma}_{11}(0) \rangle - \langle \hat{\sigma}_{mm}(0) \rangle) X_{n2,2m}(\omega_s) F_{1n,m1}(\omega_i) \\ & + g_{2n}(-\omega_s) g_{m1}(-\omega_s) g_{21}(\omega_i - \omega_s) \langle \hat{\sigma}_{11}(0) \rangle X_{n2,m1}(\omega_s) F_{1n,2m}(\omega_i) \\ & + g_{2n}(-\omega_s) g_{2m}(-\omega_s) g^*_{12}(-\omega_i + \omega_s) \langle \hat{\sigma}_{mm}(0) \rangle X_{n2,2m}(\omega_s) F_{1n,m1}(\omega_i) \\ \big]. \end{aligned} \tag{S37}$$

In this study, we assumed that the molecule is initially in its ground state. In this case, $\langle \hat{\sigma}_{nn}(0) \rangle = \delta_{1,n}$ holds, and the scattering intensity in Eq. (S37) can be written as the sum of four terms as follows.

$$S(\omega_s) = \frac{1}{\pi} \sum_{n,m} \mathrm{Re}\left[\sum_{p=1}^{4} D^{(p)}_{n,m}(\omega_i, \omega_s) Y^{(p)}_{n,m}(\omega_s) I^{(p)}_{n,m}(\omega_i)\right], \tag{S38}$$

$$\begin{pmatrix} D^{(1)}_{n,m}(\omega_i,\omega_s) \\ D^{(2)}_{n,m}(\omega_i,\omega_s) \\ D^{(3)}_{n,m}(\omega_i,\omega_s) \\ D^{(4)}_{n,m}(\omega_i,\omega_s) \end{pmatrix} = \begin{pmatrix} g_{m1}(-\omega_s)g_{n1}(\omega_i)g^*_{12}(-\omega_i+\omega_s) \\ g_{m1}(-\omega_s)g_{n1}(-\omega_s)g^*_{12}(-\omega_i+\omega_s) \\ g_{m1}(\omega_i)g_{2n}(-\omega_s)g_{21}(\omega_i-\omega_s) \\ g_{2n}(-\omega_s)g_{m1}(-\omega_s)g_{21}(\omega_i-\omega_s) \end{pmatrix}, \tag{S39}$$

$$\begin{pmatrix} I^{(1)}_{n,m}(\omega_i) \\ I^{(2)}_{n,m}(\omega_i) \\ I^{(3)}_{n,m}(\omega_i) \\ I^{(4)}_{n,m}(\omega_i) \end{pmatrix} = \begin{pmatrix} F_{m2,n1}(\omega_i) \\ F_{m2,2n}(\omega_i) \\ F_{1n,m1}(\omega_i) \\ F_{1n,2m}(\omega_i) \end{pmatrix}, \tag{S40}$$

$$\begin{pmatrix} Y^{(1)}_{n,m}(\omega_s) \\ Y^{(2)}_{n,m}(\omega_s) \\ Y^{(3)}_{n,m}(\omega_s) \\ Y^{(4)}_{n,m}(\omega_s) \end{pmatrix} = \begin{pmatrix} X_{1m,2n}(\omega_s) \\ X_{1m,n1}(\omega_s) \\ X_{n2,2m}(\omega_s) \\ X_{n2,m1}(\omega_s) \end{pmatrix}. \tag{S41}$$

Eq. (S38) provides the final expression for the TERS intensity. This expression can be evaluated by computing the near field $\boldsymbol{E}_{\mathrm{loc}}(\boldsymbol{r},\omega_i)$ and the renormalized Green's function $\boldsymbol{G}_{\mathrm{ren}}(\boldsymbol{r},\boldsymbol{r'},\omega_s)$ using classical electromagnetic analysis. In this study, $\boldsymbol{G}_{\mathrm{ren}}(\boldsymbol{r},\boldsymbol{r'},\omega_i)$ was computed by the DDA,[8] in the same manner as $\boldsymbol{E}_{\mathrm{loc}}(\boldsymbol{r},\omega_i)$. Although several approaches exist for nonresonant SERS and TERS that similarly describe scattering using Green's functions and evaluate the electromagnetic field using the DDA or related methods, the present theory can describe TERS while explicitly incorporating electronic resonance effects. Approaches that describe SERS and TERS using Green's functions and evaluate them via electromagnetic simulations such as the DDA have been reported previously.[13,14] However, the present theory is distinct in that it can describe TERS while incorporating electronic resonance effects, and in that it treats both the incident frequency and the scattered field self-consistently with the molecular response within a nonlocal-response framework. Owing to these features, our theory can describe Raman scattering mediated by electronically forbidden transitions, including multipole–multipole scattering processes. It can further capture the interference

effect by which the spatial distributions of both the FC and HT transition dipoles are transferred to the TERS maps.

The four terms in Eq. (S38) correspond to the four contributions contained in the Kramers-Heisenberg-Dirac (KHD) dispersion formula.[11] In what follows, we verify that, in the absence of metal structures and under conditions where the long-wavelength approximation holds, the scattered photon number predicted by Eq. (S38) agrees with that given by the KHD dispersion formula.

## 1.4 Relation to the KHD dispersion formula

In this section, we consider the number of scattered photons per unit time obtained from Eq. (S38) in the absence of metal structures and under conditions where the long-wavelength approximation is valid. For simplicity, we set all relaxation rates to zero. In vacuum, the mode function $\boldsymbol{f}_\eta(\boldsymbol{r})$ can be written as a plane wave as follows:

$$\boldsymbol{f}_\eta(\boldsymbol{r}) = \frac{1}{\sqrt{V}}\boldsymbol{e}_\eta e^{i\boldsymbol{k}_\eta}, \tag{S42}$$

where, $\boldsymbol{e}_\eta$ and $\boldsymbol{k}_\eta$ are the unit polarization vector and the wave vector, respectively, and V is the system volume. Under the long-wavelength approximation, the electric field can be regarded as uniform over the molecule with a constant value $\boldsymbol{E}_0$. Accordingly, the following approximation holds for $F_{nm,n'm'}(\omega_i)$:

$$F_{nm,n'm'}(\omega_i) \simeq |\boldsymbol{E}_0|^2(\boldsymbol{e}_i \cdot \boldsymbol{\mu}_{nm})(\boldsymbol{e}_i \cdot \boldsymbol{\mu}_{n'm'}), \quad \boldsymbol{\mu}_{nm} = \int d\boldsymbol{r}\boldsymbol{\mathcal{P}}_{nm(}\boldsymbol{r}), \tag{S43}$$

where $\boldsymbol{\mu}_{mn}$ is the transition dipole moment of the molecule and $\boldsymbol{e}_i$ is the unit polarization vector for incident field. This $\boldsymbol{E}_0$ can be expressed in terms of the incident photon flux $N_i$ —i.e., the number of incident photons crossing a unit area per unit time at the incident

frequency—as follows:

$$|\boldsymbol{E}_0|^2 = \frac{\hbar\omega_i N_i}{2\varepsilon_0 c}. \tag{S44}$$

Applying these approximations to Eq. (S38), and further substituting the resulting expression for $S(\omega_s)$ into Eq. (S21), we obtain the expression for the number of scattered photons emitted per unit time as follows:

$$\begin{aligned}
&\frac{d}{dt}\left\langle \hat{a}_\eta^\dagger(t)\hat{a}_\eta(t)\right\rangle \\
&= \int_0^\infty d\omega_s \sum_{n,m,\eta} \frac{\omega_i\omega_s\pi N_i}{2\varepsilon_0^2 cV}\delta(\omega_{21}-\omega_i+\omega_s)\delta(\omega_s-\Omega_\eta) \\
&\times\left\{\frac{(\boldsymbol{\mu}_{1m}\cdot\boldsymbol{e}_\eta)(\boldsymbol{\mu}_{2n}\cdot\boldsymbol{e}_\eta)(\boldsymbol{\mu}_{m2}\cdot\boldsymbol{e}_i)(\boldsymbol{\mu}_{n1}\cdot\boldsymbol{e}_i)}{(\omega_{m1}+\omega_s)(\omega_{n1}-\omega_i)} + \frac{(\boldsymbol{\mu}_{1m}\cdot\boldsymbol{e}_\eta)(\boldsymbol{\mu}_{n1}\cdot\boldsymbol{e}_\eta)(\boldsymbol{\mu}_{m2}\cdot\boldsymbol{e}_i)(\boldsymbol{\mu}_{2n}\cdot\boldsymbol{e}_i)}{(\omega_{m1}+\omega_s)(\omega_{n1}+\omega_s)}\right. \\
&\left.+\frac{(\boldsymbol{\mu}_{n2}\cdot\boldsymbol{e}_\eta)(\boldsymbol{\mu}_{2m}\cdot\boldsymbol{e}_\eta)(\boldsymbol{\mu}_{1n}\cdot\boldsymbol{e}_i)(\boldsymbol{\mu}_{m1}\cdot\boldsymbol{e}_i)}{(\omega_{n2}-\omega_s)(\omega_{m1}-\omega_i)} + \frac{(\boldsymbol{\mu}_{n2}\cdot\boldsymbol{e}_\eta)(\boldsymbol{\mu}_{m1}\cdot\boldsymbol{e}_\eta)(\boldsymbol{\mu}_{1n}\cdot\boldsymbol{e}_i)(\boldsymbol{\mu}_{2m}\cdot\boldsymbol{e}_i)}{(\omega_{n2}-\omega_s)(\omega_{m1}+\omega_s)}\right\}.
\end{aligned} \tag{S45}$$

Furthermore, The delta function $\delta(\omega_s - \Omega_\eta)$ is related to the photon density of states $D(\omega_s)$ as follows:

$$\sum_\eta \delta(\omega_s - \Omega_\eta) = D(\omega_s). \tag{S46}$$

Using this relation, and neglecting the polarization degrees of freedom, we restrict the sum over modes $\eta$ to those scattered into the solid-angle element $d\Omega$. The resulting mode sum over the delta function can then be written as follows:

$$\sum_\eta \delta(\omega_s - \Omega_\eta) = \frac{V\omega_s^2}{(2\pi c)^3}d\Omega. \tag{S47}$$

Assuming a fixed polarization vector $\boldsymbol{e}_s$ for the scattered photons, substituting Eq. (S47) into Eq. (S45) yields:

$$\frac{d}{dt}\langle \hat{a}_\eta^\dagger(t)\hat{a}_\eta(t)\rangle = \frac{\omega_i\omega_s^3 N_i d\Omega}{16\pi^2 c^4\varepsilon_0^2}\left|\sum_n\left\{\frac{(\boldsymbol{\mu}_{2n}\cdot\boldsymbol{e}_s)(\boldsymbol{\mu}_{n1}\cdot\boldsymbol{e}_i)}{(\omega_{n1}-\omega_i)}+\frac{(\boldsymbol{\mu}_{2n}\cdot\boldsymbol{e}_i)(\boldsymbol{\mu}_{n1}\cdot\boldsymbol{e}_s)}{(\omega_{n2}+\omega_i)}\right\}\right|^2. \tag{S48}$$

Eq. (S48) agrees with the result obtained from the KHD dispersion formula.[11] Based on the above discussion, the TERS theory derived in the previous section is consistent with the conventional KHD dispersion formula and can be viewed as its natural extension.

## 1.5 Herzberg-Teller (HT) expansion for transition dipole density

Using Herzberg-Teller (HT) expansion,[9] the transition dipole density entering Eq. (S7) is expanded as

$$\boldsymbol{\mathcal{P}}_{nm}(\boldsymbol{r}) = \boldsymbol{\mathcal{P}}_{nm}^{(\mathrm{FC})}(\boldsymbol{r})\,\langle v_n|v_m\rangle + \boldsymbol{\mathcal{P}}_{nm,k}^{(\mathrm{HT})}(\boldsymbol{r})\,\langle v_n|\hat{Q}_k|v_m\rangle, \tag{S49}$$

where $\hat{Q}_k$ is the normal coordinate operator of $k$–th vibrational mode, and $v_n$ denotes the number of vibrational quanta in state $n$. Franck-Condon (FC) term $\boldsymbol{P}_{nm}^{(\mathrm{FC})}(\boldsymbol{r})$ is the electronic transition dipole moment, whereas HT term $\boldsymbol{P}_{nm}^{(\mathrm{HT})}(\boldsymbol{r})$ is the component arising from the vibrationally induced perturbation. $\boldsymbol{\mathcal{P}}_{nm}^{(\mathrm{FC})}(\boldsymbol{r})$ can be evaluated from the electronic wavefunction $\psi_n(\boldsymbol{r})$ of state $n$ and the electronic wavefunction $\psi_m(\boldsymbol{r})$ of state $m$ at the equilibrium nuclear geometry. To compute an origin-independent $\boldsymbol{\mathcal{P}}_{nm}^{(\mathrm{FC})}(\boldsymbol{r})$, we adopt an approach in which the polarization is obtained from the transition current density.[15] This leads to the following expression:

$$\boldsymbol{\mathcal{P}}_{nm}^{(\mathrm{FC})}(\mathbf{r}) = \frac{e\hbar}{2m_e(\omega_m-\omega_n)}\{\psi_n^*(\boldsymbol{r})\boldsymbol{\nabla}\psi_m(\boldsymbol{r}) - (\boldsymbol{\nabla}\psi_n^*(\boldsymbol{r}))\psi_m(\boldsymbol{r})\}, \tag{S50}$$

where $e$ and $m_e$ denote the elementary charge and the electron mass, respectively. $\boldsymbol{\mathcal{P}}^{(\mathrm{HT})}_{nm,k}(\boldsymbol{r})$ is given by the derivative of the transition dipole with respect to $Q_k$. We therefore displaced the nuclei from their equilibrium positions along the vibrational mode of interest such that the corresponding normal coordinate becomes $\Delta Q_k$. Using the resulting nuclear coordinates, together with the other molecular-orbital parameters obtained from the geometry optimization at the equilibrium structure, we constructed the electronic orbitals. Substituting these orbitals into Eq. (S50), we obtained the transition dipole density $\boldsymbol{\mathcal{M}}_{nm}(\boldsymbol{r}, \Delta Q_k)$ for the displaced nuclear geometry. We then evaluated $\boldsymbol{\mathcal{P}}^{(\mathrm{HT})}_{nm,k}(\boldsymbol{r})$ from the central finite difference of $\boldsymbol{\mathcal{M}}_{nm}(\boldsymbol{r}, \Delta Q_k)$ as follows:

$$\boldsymbol{\mathcal{P}}^{(\mathrm{HT})}_{nm,k}(\boldsymbol{r}) = \frac{\boldsymbol{\mathcal{M}}_{nm}(\boldsymbol{r} : \Delta Q_k) - \boldsymbol{\mathcal{M}}_{nm}(\boldsymbol{r} : -\Delta Q_k)}{2\Delta Q_k}. \tag{S51}$$

We now discuss the FC overlap $\langle v_n | v_m \rangle$. Within the linear-coupling model,[16,17] the FC overlap for $(v_n \geq v_m)$ can be written as follows.

$$\langle v_n | v_m \rangle = e^{-d^2/2} \sqrt{\frac{v_m!}{v_n!}} (-d)^{(v_n - v_m)} L^{(v_n - v_m)}_{v_m}(d^2), \tag{S52}$$

For $(v_n < v_m)$, the Franck–Condon overlap is given by the following expression.

$$\langle v_n | v_m \rangle = e^{-d^2/2} \sqrt{\frac{v_n!}{v_m!}} d^{(v_m - v_n)} L^{(v_m - v_n)}_{v_n}(d^2), \tag{S53}$$

with $d$ $k$–th normal mode dimensionless displacement and $L^{\alpha}_{\beta}$ the associated Laguerre polynomial. After performing vibrational analyses for the molecule in the ground and excited states via quantum-chemical calculations $d$ can be obtained from these results using the FCclasses3 package.[18]

$\hat{Q}_k$ can be written in terms of the vibrational creation ($\hat{b}_k$) and annihilation ($\hat{b}^{\dagger}_k$) operators

as follows.

$$\hat{Q}_k = \sqrt{\frac{\hbar}{2\omega_k}}(\hat{b}_k + \hat{b}_k^\dagger), \tag{S54}$$

where $\omega_k$ is $k$–th mode vibrational frequency. Accordingly, the factor appearing in the second term on the right-hand side of Eq. (S49) can be written as follows.

$$\langle v_n|\hat{Q}_k|v_m\rangle = \sqrt{\frac{\hbar}{2\omega_k}}(\sqrt{v_n}\langle v_n - 1|v_m\rangle + \sqrt{v_n + 1}\langle v_n + 1|v_m\rangle). \tag{S55}$$

## 1.6 Approximations for Nonresonant Raman Scattering

In the main text, we validated the present theory by comparing its predictions with the TERS measurements and calculations on pentacene previously reported by Xu et al..[19] In contrast, the experiments by Xu et al. probed nonresonant Raman scattering, and were therefore performed under conditions different from those considered in the main text, which focuses primarily on resonant Raman scattering. Accordingly, we applied the nonresonant Raman approximation to Eq. S38 and performed calculations under off-resonant excitation, i.e., with an incident photon energy detuned from the molecular excitation energy. Here, although multiple electronic excitations can, in principle, contribute to nonresonant Raman scattering, we assume that the contribution mediated by the first excited state—typically having the largest oscillator strength—is dominant, and we therefore restrict the following discussion to this component.

In nonresonant Raman scattering, an approximation commonly referred to as Placzek's polarizability theory is generally applicable.[20] When the incident photon energy is sufficiently detuned from the molecular excitation energy, the vibrational-energy term and relaxation rates in the energy denominator can be neglected. Here, Eq. (S38) contains a sum over molecular states based on the Born–Oppenheimer approximation;[6] however, if the electronic contribution is restricted to the first excited state, this sum reduces to a sum over vibrational

levels. Since the vibrational contributions in the energy denominator are neglected, the completeness relation over vibrational states can be applied. Moreover, because the transition dipole density $\boldsymbol{\mathcal{P}}_{nm}(\boldsymbol{r})$ is assumed to be real-valued here, the relation $(\boldsymbol{\mathcal{P}}_{nm}(\boldsymbol{r}) = \boldsymbol{\mathcal{P}}_{mn}(\boldsymbol{r})$ can be used to simplify the expression. This yields the following expression for the nonresonant Raman scattering intensity $S(\omega_s)$

$$S(\omega_s) = \frac{1}{\pi}\mathrm{Re}[g_{21}(\omega_i - \omega_s)\sum_p^4 K^{(p)}(\omega_i,\omega_s)\sum_q^4 J^{(q)}(\omega_i,\omega_s)], \tag{S56}$$

where

$$\begin{pmatrix} K^{(1)}(\omega_i,\omega_s) \\ K^{(2)}(\omega_i,\omega_s) \\ K^{(3)}(\omega_i,\omega_s) \\ K^{(4)}(\omega_i,\omega_s) \end{pmatrix} = \begin{pmatrix} \hbar^{-2}(-\omega_{\mathrm{ex}} - \omega_s)^{-1}(\omega_{\mathrm{ex}} - \omega_i)^{-1} \\ \hbar^{-2}(-\omega_{\mathrm{ex}} - \omega_s)^{-1}(\omega_{\mathrm{ex}} + \omega_s)^{-1} \\ \hbar^{-2}(\omega_{\mathrm{ex}} - \omega_i)^{-1}(-\omega_{\mathrm{ex}} + \omega_s)^{-1} \\ \hbar^{-2}(-\omega_{\mathrm{ex}} + \omega_s)^{-1}(\omega_{\mathrm{ex}} + \omega_s)^{-1} \end{pmatrix}, \tag{S57}$$

$$\begin{aligned} &J^{(1)}(\omega_i,\omega_s) \\ &= \frac{\hbar}{2\omega_{\mathrm{v}}}\left(\int d\boldsymbol{r}_1 \boldsymbol{\mathcal{P}}^{(S_1)}_{\mathrm{FC}}(\boldsymbol{r}_1)\cdot \boldsymbol{E}_{\mathrm{loc}}(\boldsymbol{r}_1,-\omega_i)\right)\left(\int d\boldsymbol{r}_2 \boldsymbol{\mathcal{P}}^{(S_1)}_{\mathrm{FC}}(\boldsymbol{r}_2)\cdot \boldsymbol{E}_{\mathrm{loc}}(\boldsymbol{r}_2,\omega_i)\right) \\ &\cdot \left(\iint d\boldsymbol{r}_3 d\boldsymbol{r}_4 \boldsymbol{\mathcal{P}}^{(S_1)}_{\mathrm{HT}}(\boldsymbol{r}_3)\boldsymbol{G}_{\mathrm{ren}}(\boldsymbol{r}_3,\boldsymbol{r}_4,\omega_s)\boldsymbol{\mathcal{P}}^{(S_1)}_{\mathrm{HT}}(\boldsymbol{r}_4)\right), \end{aligned} \tag{S58}$$

$$\begin{aligned} &J^{(2)}(\omega_i,\omega_s) \\ &= \frac{\hbar}{2\omega_{\mathrm{v}}}\left(\int d\boldsymbol{r}_1 \boldsymbol{\mathcal{P}}^{(S_1)}_{\mathrm{HT}}(\boldsymbol{r}_1)\cdot \boldsymbol{E}_{\mathrm{loc}}(\boldsymbol{r}_1,-\omega_i)\right)\left(\int d\boldsymbol{r}_2 \boldsymbol{\mathcal{P}}^{(S_1)}_{\mathrm{HT}}(\boldsymbol{r}_2)\cdot \boldsymbol{E}_{\mathrm{loc}}(\boldsymbol{r}_2,\omega_i)\right) \\ &\cdot \left(\iint d\boldsymbol{r}_3 d\boldsymbol{r}_4 \boldsymbol{\mathcal{P}}^{(S_1)}_{\mathrm{FC}}(\boldsymbol{r}_3)\boldsymbol{G}_{\mathrm{ren}}(\boldsymbol{r}_3,\boldsymbol{r}_4,\omega_s)\boldsymbol{\mathcal{P}}^{(S_1)}_{\mathrm{FC}}(\boldsymbol{r}_4)\right), \end{aligned} \tag{S59}$$

$$J^{(3)}(\omega_i, \omega_s)$$
$$= \frac{\hbar}{2\omega_{\mathrm{v}}} \left( \int d\boldsymbol{r}_1 \boldsymbol{\mathcal{P}}_{\mathrm{HT}}^{(S_1)}(\boldsymbol{r}_1) \cdot \boldsymbol{E}_{\mathrm{loc}}(\boldsymbol{r}_1, -\omega_i) \right) \left( \int d\boldsymbol{r}_2 \boldsymbol{\mathcal{P}}_{\mathrm{FC}}^{(S_1)}(\boldsymbol{r}_2) \cdot \boldsymbol{E}_{\mathrm{loc}}(\boldsymbol{r}_2, \omega_i) \right)$$
$$\cdot \left( \iint d\boldsymbol{r}_3 d\boldsymbol{r}_4 \boldsymbol{\mathcal{P}}_{\mathrm{FC}}^{(S_1)}(\boldsymbol{r}_3) \boldsymbol{G}_{\mathrm{ren}}(\boldsymbol{r}_3, \boldsymbol{r}_4, \omega_s) \boldsymbol{\mathcal{P}}_{\mathrm{HT}}^{(S_1)}(\boldsymbol{r}_4) \right), \tag{S60}$$

$$J^{(4)}(\omega_i, \omega_s)$$
$$= \frac{\hbar}{2\omega_{\mathrm{v}}} \left( \int d\boldsymbol{r}_1 \boldsymbol{\mathcal{P}}_{\mathrm{FC}}^{(S_1)}(\boldsymbol{r}_1) \cdot \boldsymbol{E}_{\mathrm{loc}}(\boldsymbol{r}_1, -\omega_i) \right) \left( \int d\boldsymbol{r}_2 \boldsymbol{\mathcal{P}}_{\mathrm{HT}}^{(S_1)}(\boldsymbol{r}_2) \cdot \boldsymbol{E}_{\mathrm{loc}}(\boldsymbol{r}_2, \omega_i) \right)$$
$$\cdot \left( \iint d\boldsymbol{r}_3 d\boldsymbol{r}_4 \boldsymbol{\mathcal{P}}_{\mathrm{HT}}^{(S_1)}(\boldsymbol{r}_3) \boldsymbol{G}_{\mathrm{ren}}(\boldsymbol{r}_3, \boldsymbol{r}_4, \omega_s) \boldsymbol{\mathcal{P}}_{\mathrm{FC}}^{(S_1)}(\boldsymbol{r}_4) \right). \tag{S61}$$

Here, $\omega_{\mathrm{ex}}$ denotes the energy of the first electronic excited state, $\boldsymbol{\mathcal{P}}_{\mathrm{FC}}^{(S_1)}(\boldsymbol{r})$ and $\boldsymbol{\mathcal{P}}_{\mathrm{HT}}^{(S_1)}(\boldsymbol{r})$ are the FC and HT transition dipole densities associated with the transition to the first excited state. This approximation simplifies the description of the intermediate states in nonresonant Raman scattering, thereby facilitating the calculations.

# 2 Applicability and limitations

## 2.1 Temperature and environmental effects

In this study, Eq. (S38) is derived for a cryogenic/UHV condition, where the molecule is assumed to remain predominantly in its ground state. For finite-temperature SERS/TERS, however, Eq. (S37) can be used instead of Eq. (S38), because Eq. (S37) explicitly includes the populations of the relevant electronic/vibrational levels. In practice, environmental effects can be incorporated mainly through (i) the initial level populations and (ii) relaxation/pure-dephasing parameters (e.g., $\gamma_{\mathrm{damp}}$ and $\gamma_{\mathrm{phase}}$), which determine the effective linewidths entering the response.

At elevated temperature, thermal population of vibrationally excited levels modifies the Stokes/anti-Stokes balance, while increased dephasing and spectral diffusion broaden reso-

nant features. These effects reduce resonant selectivity and can wash out spatial contrast associated with electronically forbidden resonant pathways. Under non-UHV/ambient conditions, additional averaging due to molecular diffusion and fluctuating adsorption geometries, as well as changes in the local dielectric environment, can further smear single-molecule TERS maps.

Nevertheless, the same framework remains useful for analyzing more general TERS/SERS spectra (and, when meaningful, images) by using temperature-/environment-dependent populations, linewidth parameters, and effective environmental screening.

## 2.2 Strong plasmon–molecule coupling and model scope

The present formulation does not introduce an external plasmon–exciton coupling constant (e.g., a phenomenological parameter $g$). Instead, the plasmon–molecule interaction is determined by the geometric configuration (molecular position/orientation and nanogap/tip–substrate geometry) together with the electromagnetic response encoded in the Green tensor and the resulting self-consistent field. Therefore, no additional coupling parameter is required to describe cases with stronger plasmon–molecule backaction within the present framework. If the junction geometry and material parameters yield sufficiently strong self-consistent backaction, hybridized plasmon–molecule resonances can emerge from the same set of equations.

In such cases, the main change is in the interpretation of the Raman spectra and images: multiple hybridized resonances may contribute, and the resulting spectral structures and spatial maps can become richer. A careful analysis may then require considering state mixing and contributions from multiple hybridized resonances.

A closely related nonlocal, self-consistent strong-coupling analysis has been demonstrated for single-molecule tip-enhanced photoluminescence (TEPL), where hybridized states and Rabi splitting emerge without introducing an external coupling constant.[7]

## 2.3 Scope with respect to population-driven emission / luminescence channels

The present formulation targets vibronic Raman scattering treated as a second-order (two-photon) process: intermediate electronic/vibronic states enter through the Raman amplitudes (including FC/HT contributions) with phenomenological dephasing parameters, and the observable is the Stokes-frequency photon flux generated by the field-driven Raman polarization. Thus, the present framework is formulated to include the Raman-scattering contribution only, and does not include terms corresponding to population-driven emission/luminescence channels. In principle, such population-driven emission channels could be incorporated in an extended framework that includes real excited-state populations and relaxation/thermalization dynamics (e.g., rate-equation or master-equation dynamics), for example by combining the present Raman-scattering formulation with the nonlocal, self-consistent emission/luminescence framework developed in Ref.;[7] such an extension is a natural and important direction for future work. Experimentally, Raman and luminescence contributions can often be distinguished by their spectral behavior: Raman peaks track the excitation wavelength via the Raman shift, whereas luminescence is governed by emitting-state energies. In cases where spectral overlap is substantial, additional diagnostics (e.g., linewidth/lineshape, power dependence, or time-resolved behavior) may be required.

# 3 Computational Details

Electronic structure calculations were performed with Gaussian 16,[21] using B3LYP functional[22] and the 6-31G** basis set.[23] Excited states were computed using TD–DFT with the Tamm–Dancoff approximation.[24] Transition dipole densities were constructed from Kohn–Sham orbitals for all excited states up to $S_2$. In addition, vibrational analyses were performed using Gaussian 16 for the ground state obtained by DFT and for the excited states up to $S_2$

obtained by TD-DFT.

The cavity field and renormalized Green's function were computed using DDA.[8] Material dispersion was modeled by the Drude–two–critical–points model.[25] The mesh size was set to 0.1 nm. The tip position was scanned laterally in the $xy$ plane to construct TERS maps.

In our Raman scattering calculations, we assume that the molecule is in its electronic ground state in the initial state, i.e., $\langle \hat{\sigma}_{nn}(0) \rangle = \delta_{1,n}$. Furthermore, we set the relaxation rate between states 1 and 2 to a value corresponding to a linewidth of 10 cm$^{-1}$. The other relaxation rates were set to $2 \times 10^{-5}$ eV. Based on the TDDFT results, we set the excitation energies of the S1 and S2 states of pentacene to 2.0182 eV and 2.7322 eV, respectively. For the $S_1$-resonant TERS calculations of the v1–v6 modes, the incident photon energy was set to 2.0478 eV (605.44 nm), 2.0654 eV (600.28 nm), 2.0752 eV (597.45 nm), 2.0780 eV (596.65 nm), 2.1321 eV (581.51 nm), and 2.1454 eV (577.88 nm), respectively. These values were obtained by adding the vibrational energy to the excitation energy of the $S_1$ state. For the $S_2$-resonant TERS calculations, the incident photon energy was set to 2.7618 eV (448.92 nm), 2.7794 eV (446.08 nm), 2.7892 eV (444.51 nm), 2.7937 eV (443.80 nm), 2.8461 eV (435.63 nm), and 2.8595 eV (433.559 nm) for v1–v6, respectively. These values likewise correspond to the sum of the $S_2$ excitation energy and the vibrational energy. All nonresonant TERS calculations were performed under an incident photon energy of 2.6 eV (477 nm).

We also set the excitation energies and Franck–Condon factors of the deficient–pentacene to be identical to those of pristine pentacene. In deficient–pentacene, the loss of a hydrogen molecule leaves a singly occupied molecular orbital (SOMO) state, which is formed by removing one electron from the HOMO of pristine pentacene. We treat the transition from this SOMO to the LUMO as the first excited state ($S_1$) of deficient–pentacene. The SOMO and LUMO of deficient–pentacene, as well as the structure of the FC transition dipole density associated with the SOMO–LUMO transition, are shown in Figs. S1(a)–(c), respectively.

In our TERS calculations, by taking into account the symmetry of the system, we assumed that the scattering intensity at the tip coordinate $(x, y)$ is identical to that at $(x, -y)$.

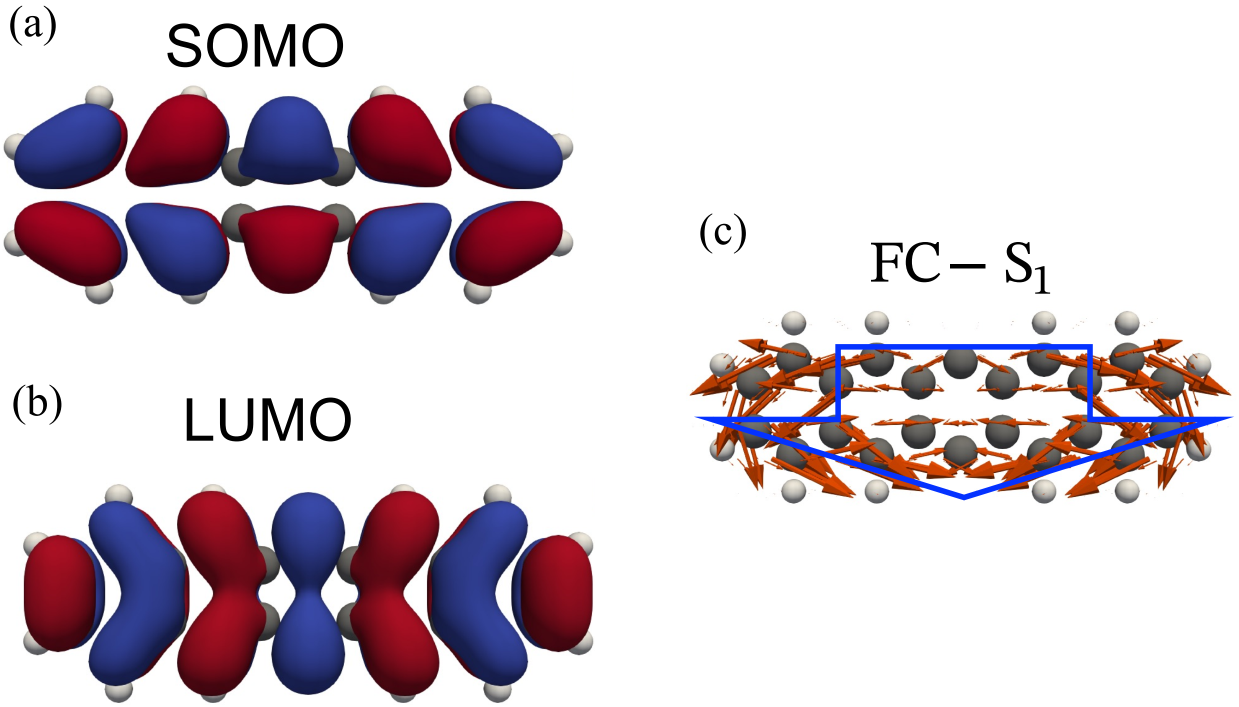


Figure S1: (a)(b) Singly occupied molecular orbital (SOMO) and lowest unoccupied molecular orbital (LUMO) of pentacene molecule missing the two central hydrogen atoms (deficient–pentacene), respectively. (c) Calculated distribution of transition dipole moments between SOMO and LUMO. The orange arrows represent the dipole moment at each point. The blue arrows indicate the overall orientation of the transition dipole moment for the entire molecule.

Similarly, for the quantity $X_{nm,n'm'}(\omega)$, we assumed that its value at the tip position $(x, y)$ is identical to that at $(-x, y)$. Based on these considerations, for $X_{nm,n'm'}(\omega)$, we avoided redundant calculations by evaluating the quantities only at one of the positions and then assigning the obtained values to the other position.

# 4 Non-normalized TERS signal levels and detectability estimate

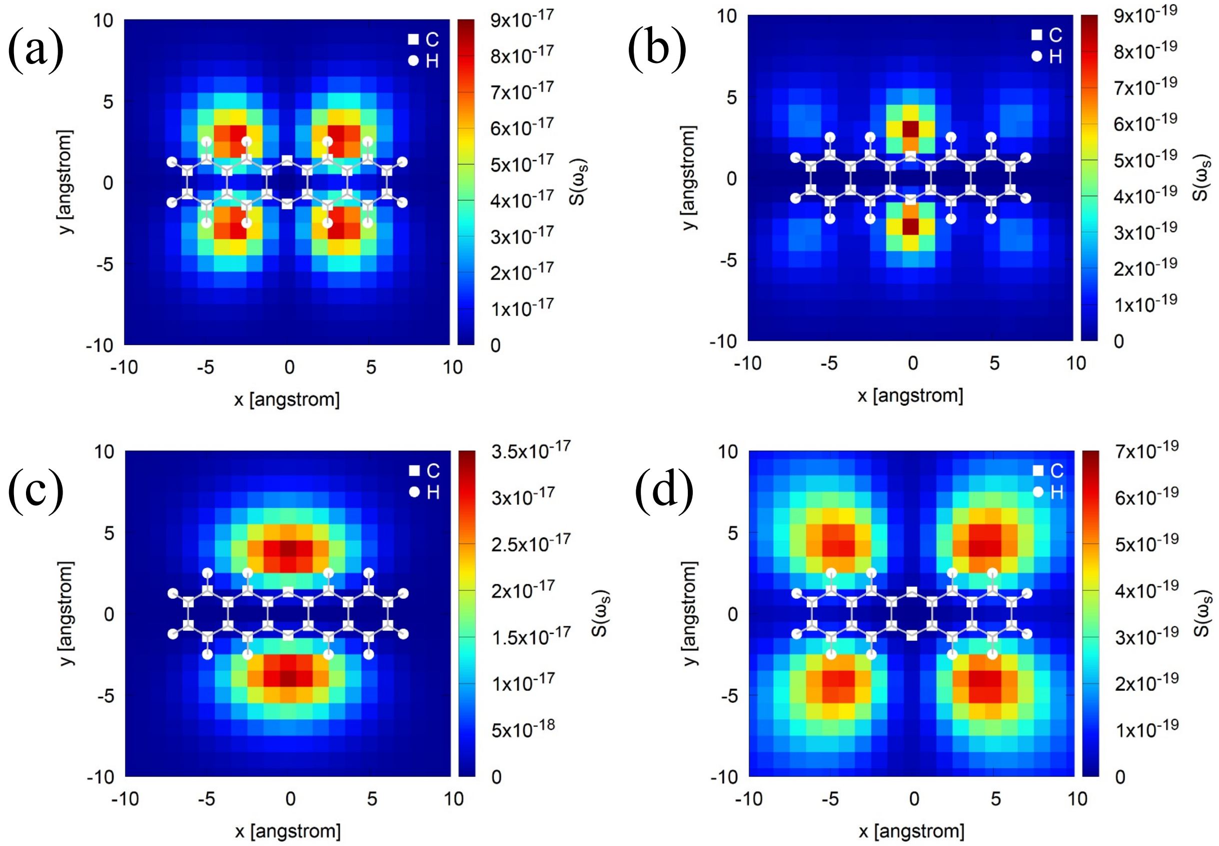


Figure S2: (a)-(d) Calculated nonresonant TERS maps for v1–v4 of the deficient–pentacene molecule, with $S(\omega_s)$ displayed directly (without normalization).

Here, we discuss non-normalized TERS signal levels, which were shown in normalized form in the main text, together with an order–of–magnitude detectability estimate. The intensities reported here are based on an incident intensity of 100 W/cm$^2$ , which is a typical excitation intensity used in single-molecule TERS experiments.[19,26] Note that $S(\omega_s)$ is a dimensionless quantity and does not itself represent the photon flux. As indicated by Eq. (S21), the photon flux is obtained by multiplying $S(\omega_s)$ by $d\omega$, which corresponds to the

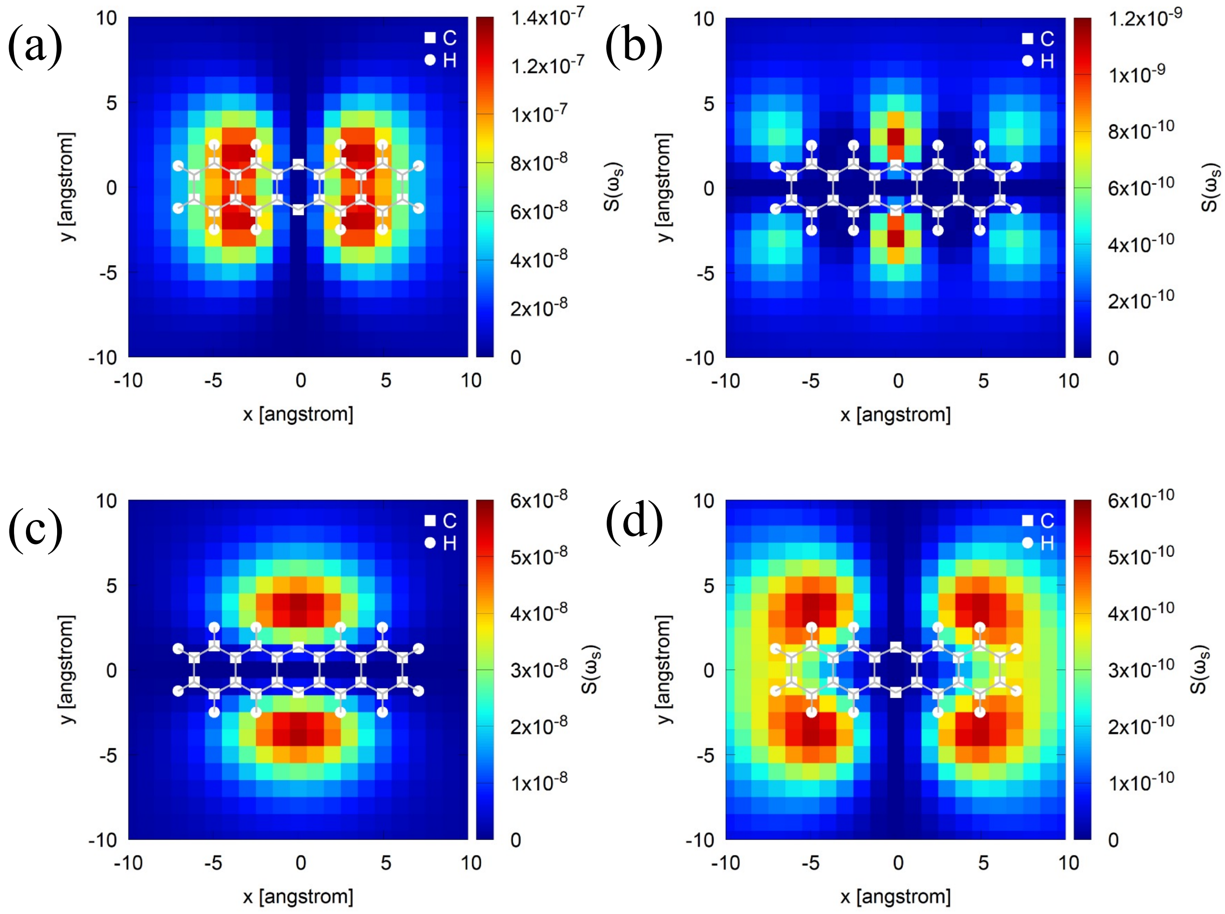


Figure S3: (a)-(d) Calculated $S_1$–resonant TERS maps for v1–v4 of the deficient–pentacene molecule, with $S(\omega_s)$ displayed directly (without normalization).

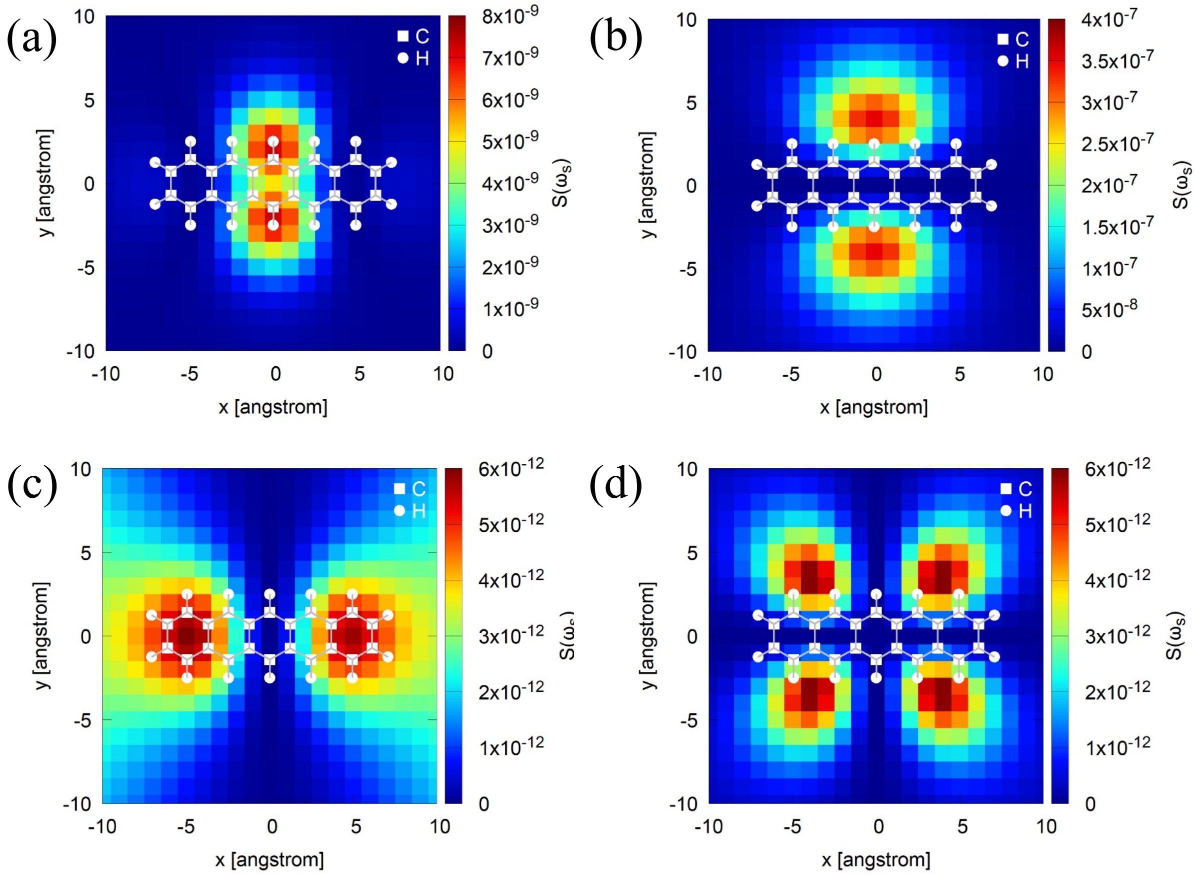


Figure S4: Calculated TERS maps of the pentacene molecule with $S(\omega_s)$ shown directly (i.e., without normalization). (a)(b) Calculated $S_1$–resonant TERS maps for v5 and v6, respectively. (c)(d) Calculated $S_2$–resonant TERS maps for v5 and v6, respectively.

spectral resolution. In Figs. S2(a)-(d), we present the calculated nonresonant TERS maps for the v1–v4 vibrational modes of deficient–pentacene, with $S(\omega_s)$ shown as–is (i.e., without normalization). These TERS intensities are on the order of $S(\omega_s) \sim 10^{-17}$ –$10^{-19}$, and among v1–v4 the weakest signal is obtained for v4, the only in–plane vibrational mode in this set. In contrast, for the $S_1$–resonant TERS of v1–v4, we show the calculated TERS maps together with the corresponding absolute values of $S(\omega_s)$ in Figs. S3(a)–(d), respectively. These results indicate that $S(\omega_s)$ takes values on the order of $10^{-8}$–$10^{-10}$, with particularly small values for v4, which corresponds to an in-plane vibration. Interestingly, Figs. S2 and S3 show that the relative ordering of the TERS intensities for v1–v4 remains unchanged between the nonresonant and $S_1$–resonant cases. We also note that, when comparing the nonresonant and $S_1$–resonant TERS maps for v1–v4, the images for each mode exhibit essentially the same nodal structures in both cases, although differences are observed in, for example, the spatial extent of the patterns.

We also show the calculated TERS maps for pristine pentacene in Fig. S4, with $S(\omega_s)$ presented without normalization. Figures S4(a)(b) show the $S_1$-resonant TERS maps for the v5 and v6 modes, respectively, whereas Figs. S4(c)(d) show the corresponding $S_2$-resonant TERS maps for v5 and v6, respectively. The $S_2$-resonant TERS intensity mediated by the electronically forbidden transition is the smallest, being on the order of $S(\omega_s) \sim 10^{-12}$ for both v5 and v6.

Because the metallic tip used in our calculations is extremely small (10 nm in diameter), the resulting TERS intensity is correspondingly weak. We therefore estimate the photon flux that would be obtained when using a metal structure with a size comparable to that employed in typical experimental conditions. Yamanishi et al. investigated how the photoinduced force between the sample (quantum dots) and the tip in photoinduced force microscopy (PiFM) depends on the tip size (see Supplementary Note 13 of Ref.[ 27]). According to this study, increasing the tip diameter from 19 nm to 60 nm (a tip size used in practical PiFM measurements) enhances the photoinduced force by a factor of 60. It was further shown that,

when the elongated geometry of a realistic tip (rather than a sphere) is taken into account, the photoinduced force is enhanced by an additional factor of about three. This implies that, for a realistic metal structure, the photoinduced force can be approximately 180 times larger than that predicted for a 19-nm-diameter spherical tip. Here, noting that the photoinduced force scales with the squared electric-field amplitude and that, in Raman scattering, an additional enhancement of the scattered field also contributes, we can roughly estimate that the scattering intensity observed in an actual measurement could be about $180^2 = 3.24 \times 10^4$ times larger than the scattering intensity obtained in our present TERS calculations. On this basis, we estimate the scattering intensity that the weakest signal—namely the $S_2$-resonant TERS—would yield in an actual measurement with a spectral resolution of 1 $\mathrm{cm}^{-1}$. A linewidth of 1 $\mathrm{cm}^{-1}$ corresponds to approximately $(3 \times 10^{10})$ Hz in frequency units. Then, the expected scattered photon flux $n$ in the measurement can be estimated as

$$n \sim S(\omega_s) \times 10^{10} \times 10^4 \ [\mathrm{s}^{-1}] \sim 10^2 \ [\mathrm{s}^{-1}]. \tag{S62}$$

In other words, even the weakest signal—the $S_2$-resonant TERS—would emit on the order of a few hundred photons per unit time, and is therefore expected to provide an intensity that is readily detectable. For context, we also note that the non-resonant calculation used for the Fig. 3 comparison in the main text (with the experimentally reported TERS image in the Science paper) yields a weaker signal level than the resonant forbidden-pathway case discussed above. The corresponding non-resonant image and its signal level are presented in Section 4. The fact that the experimentally reported image is observed provides an additional consistency check for the detectability estimate given here.

## 5 Calculated TERS images with only the $z$-dipole component

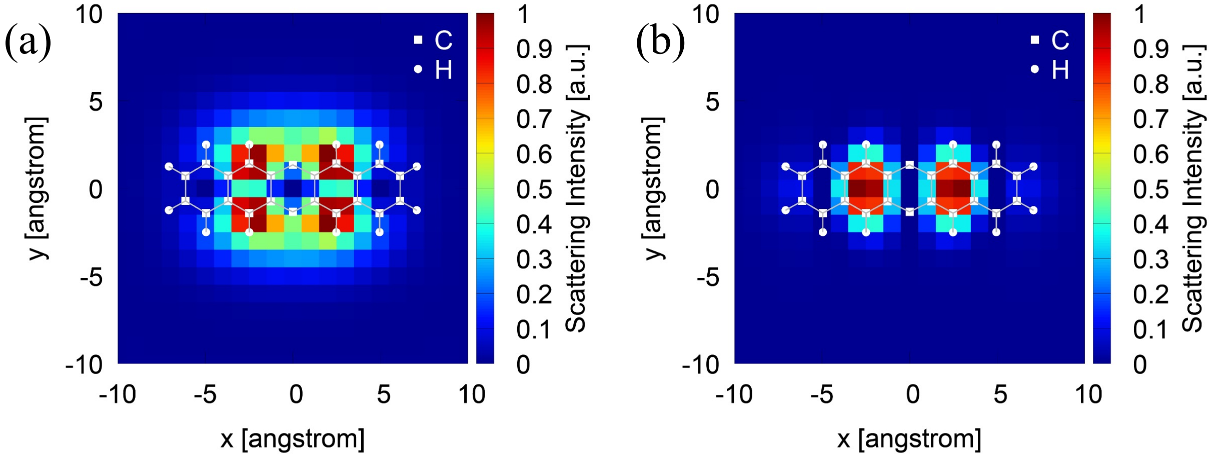


Figure S5: (a)(b) Calculated nonresonant and $S_1$–resonant ($\omega_i = \omega_{S_1} + \omega_\mathrm{v}$) TERS images of v4 of deficient–pentacene, respectively. However, in all of these calculations, the in-plane components of the transition dipole densities appearing in Eqs. (S38) and (S56) were neglected.

We performed nonresonant and $S_1$–resonant TERS calculations for deficient-pentacene. However, to maintain consistency with the simulations in the previous study,[19] we deliberately neglected all in–plane components of the transition dipoles appearing in Eqs. (S38) and (S56) when computing the TERS intensity, following the same calculation conditions. Here, we focus on the in–plane mode v4. The calculated nonresonant TERS map for v4 is shown in Fig. S5(a). This result shows that the spatial pattern in the TERS map is highly localized to regions very close to the molecule. This result is in very close agreement with the previous simulation results.[19] Moreover, regarding the spatial extent of the pattern, the calculations reported in the main text—where all in-plane components are included–reproduce the experimental observations more closely than the present results. Taken together, these findings indicate that the in–plane components of the transition dipole, which are often ne-

glected in TERS calculations, in fact play a crucial role. Similarly, Fig. S5(b) shows that the $S_1$–resonant TERS map, when calculated while neglecting the in–plane components, also exhibits a pattern that is highly localized to positions very close to the molecule. These results indicate that the in–plane components of the transition dipole are important in both nonresonant and resonant TERS.

## 6 TERS maps of singly dehydrogenated pentacene

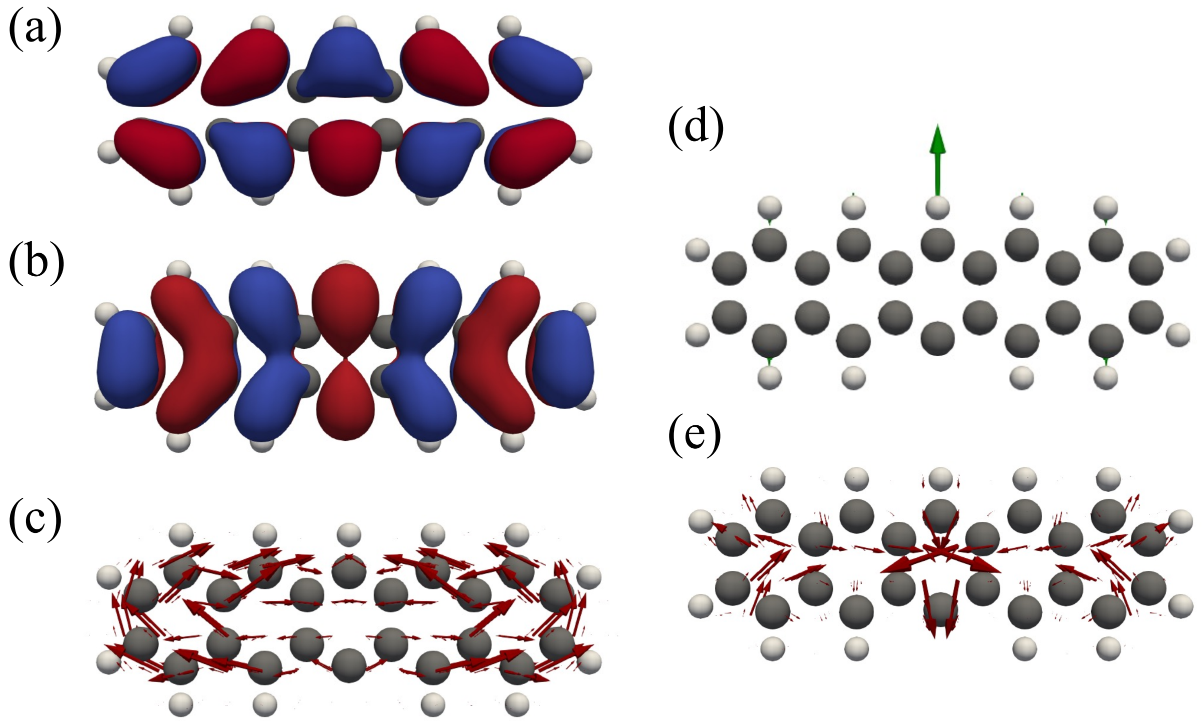


Figure S6: (a)(b) Molecular orbitals for the SOMO and LUMO of singly dehydrogenated pentacene. (c) Spatial distribution of the FC transition–dipole density associated with the $S_1$ excitation. (d) Vibrational pattern of a C–H stretching mode at 3179 $cm^{-1}$. (e) Spatial distribution of the HT transition–dipole density induced by the 3179 $cm^{-1}$ mode.

In this section, we analyze calculated TERS maps of singly dehydrogenated pentacene (i.e., pentacene with one missing hydrogen atom), motivated by the $\beta$ configuration reported by Xu *et al.*[19] Previous analyses suggest that substrate interaction and the resulting distortion can introduce a slight tilt/twist of the adsorbed molecule.[19] Here we therefore

use a “tilt-benchmark” approach: we first compute vibrational modes and transition dipoles for the isolated molecule, and then assess how a tilt-induced reorientation of the transition dipoles redistributes the simulated TERS contrast. A fully substrate-explicit treatment (adsorbed, distorted geometry and the corresponding transition densities) is in principle feasible within our framework, but it is computationally demanding and is left for future work; the present benchmark is intended to provide complementary, physically transparent trends that aid interpretation of the experimental asymmetry.

Previous work suggested that, when acquiring TERS maps in the C–H stretching region, the adsorption geometry on the metal substrate makes the vibration of the C–H bond connected to the central benzene ring contribute dominantly; consequently, scattering was observed to originate only from the region where this C–H bond remains intact.[19] We therefore focus on a C–H stretching mode in which this specific C–H bond exhibits a particularly large vibrational amplitude. The SOMO and LUMO are shown in Fig. S6(a) and Fig. S6(b), respectively. Dehydrogenation leaves an unpaired electron, resulting in a singly occupied molecular orbital (SOMO) derived from the frontier orbitals of pristine pentacene. For simplicity, we assume that the lowest optically active excitation can be described as a transition from the SOMO to the LUMO. The FC transition–dipole density associated with this excitation is shown in Fig. S6(c). We focus on a C–H stretching mode with a wavenumber of 3179 $\mathrm{cm}^{-1}$. As shown in Fig. S6(d), this mode exhibits a vibrational pattern in which the relevant C–H bond on the central benzene ring vibrates particularly strongly. The HT transition–dipole density induced by this vibration is shown in Fig. S6(e). Overall, the distribution exhibits a net moment along the molecular short axis; reflecting the short-axis asymmetry introduced by the hydrogen deficiency, the HT transition–dipole density likewise becomes asymmetric.

Assuming the molecular excitation energy to be 2.0182 eV (as in pristine pentacene), we performed non-resonant TERS calculations at an incident photon energy of 2.6 eV. The computational model, including the metal geometry and the molecular configuration, is the

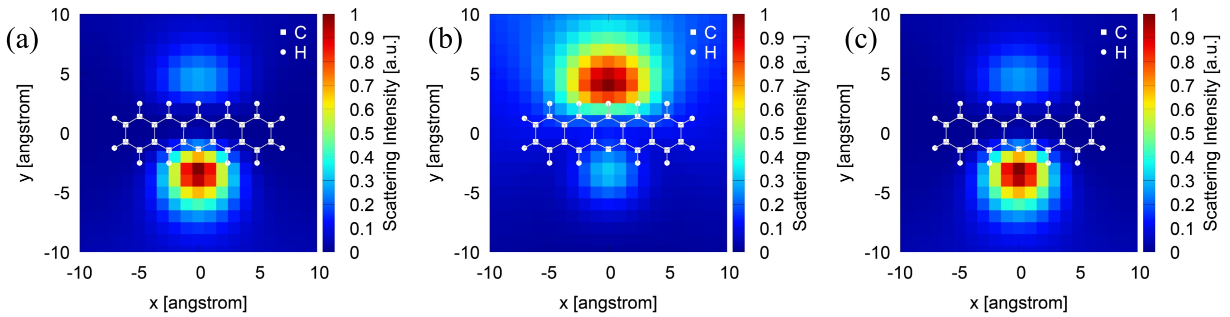


Figure S7: (a)(b) TERS maps calculated without and with the tilt-induced correction to the transition-dipole orientation, respectively. (c) TERS map calculated from the tilt-corrected transition dipole, with its $z$ component intentionally neglected.

same as that used in the main text. A previous study suggested that the central benzene ring tilts such that the side retaining the C–H bond is elevated, whereas the carbon atom where the bond is broken is lowered, resulting in a vertical displacement of 0.44 Å between the upper and lower ends of the benzene ring.[19] Using this displacement and the optimized molecular geometry, we estimated the corresponding tilt angle and then obtained transition dipoles for a configuration in which the molecule is rotated by approximately 6.53° about its long axis, such that the carbon atom with the broken C–H bond is displaced downward. We then computed and compared the TERS maps with and without this tilt correction to assess its impact. In the present calculations, the effect of the tilt is incorporated only through the orientation of the transition dipoles; geometric changes in the molecular position are not included, and the tip height is kept fixed. In the experiments, the tip was controlled in constant-current STM mode while the TERS maps were acquired, so the molecule–tip distance was kept as constant as possible.[19] This benchmark treatment is therefore intended to capture the leading trend associated with a tilt-induced reorientation relative to the tip axis.

Figure S7(a) and Fig. S7(b) show the calculated TERS maps obtained without and with the tilt-induced correction to the transition-dipole orientation, respectively. Without the tilt correction, the scattering is enhanced on the side where the C–H bond is broken. With the tilt correction, the enhanced scattering shifts to the side where the C–H bond remains,

in qualitative agreement with the trend reported in Ref.[19] This change is attributed to the tilt-induced modification of the $z$ component (the component along the tip axis) of the transition dipole. Indeed, as shown in Fig. S7(c), even when the calculation is based on transition dipoles corrected for the tilt, neglecting their $z$ component yields a result similar to the uncorrected calculation.

These results highlight that the submolecular TERS contrast can be highly sensitive to a tilt-induced reorientation of the transition dipoles and, in this sense, provide a complementary and physically transparent interpretation of the experimental asymmetry for singly dehydrogenated pentacene.[19] While the electronic and vibrational states of an adsorbed molecule may differ from those of the isolated molecule, the pronounced sensitivity to the out-of-plane ($z$) component identified here offers a clear mechanism by which adsorption-induced distortion can qualitatively redistribute the observed contrast.

# 7 Role of self-consistent Stokes-side electrodynamics: control calculations

To assess which ingredients are essential for the parity-sensitive contrast mechanism discussed in the main text, we perform an ablation-type comparison in which the vibronic Raman physics is kept fixed while only the treatment of the Stokes-side electromagnetic response is simplified. Specifically, we compare the full self-consistent theory used in the main text with a non-self-consistent single–cavity-mode-type model. The purpose is to test whether the qualitative map symmetry associated with the electronically forbidden-resonance (HT-related) pathway is preserved when the Stokes-side response is not treated self-consistently.

In our theory, contributions from many cavity modes are incorporated through a self-consistent evaluation of the near field and the renormalized Green tensor. Accordingly,

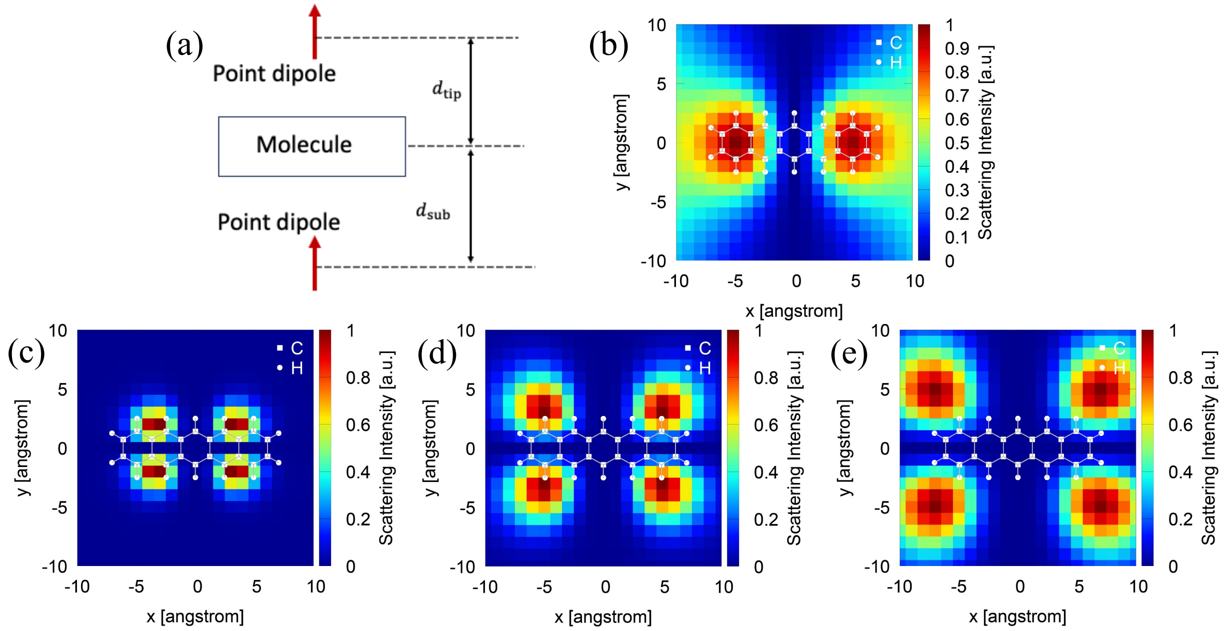


Figure S8: (a) Schematic of a non-self-consistent TERS model based on Eq. (S63), in which the metal response is represented by point dipoles. (b) Calculated $S_2$-resonant TERS map for v5 of pentacene obtained in the main text (full self-consistent theory). (c)–(e) Calculated $S_2$-resonant TERS maps for v5 obtained from Eq. (S63) for $(D_{\rm tip}, D_{\rm sub}) = (0.5\ \rm nm, 1.5\ nm)$, $(1.0\ \rm nm, 2.0\ nm)$, and $(1.5\ \rm nm, 2.5\ nm)$, respectively.

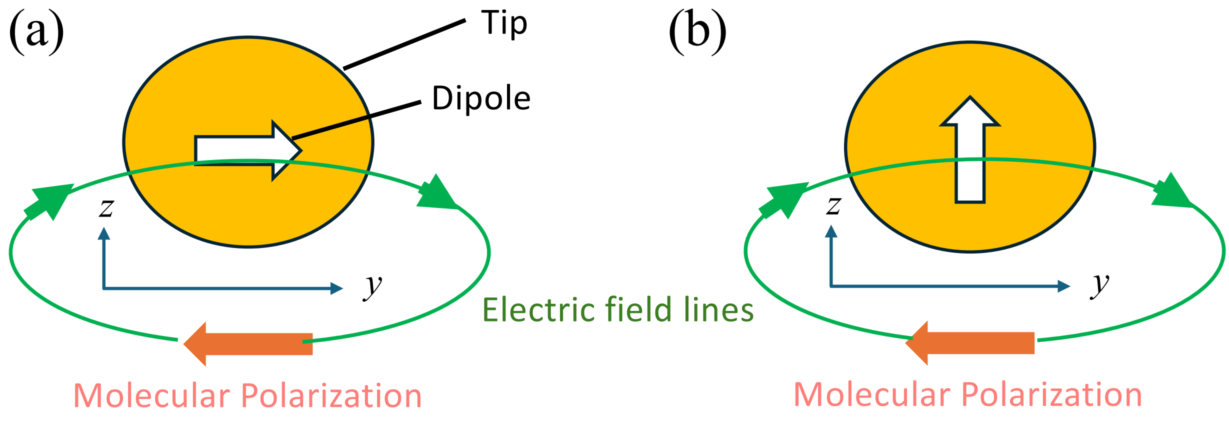


Figure S9: Schematic illustrations of the molecule–metal interaction in (a) the self-consistent calculation and (b) the non-self-consistent calculation.

depending on the relative geometry between the molecule and the metal, our framework can output TERS molecular images while accounting for the mutual influence of the optical responses of the molecule and the metal. Such effects cannot be captured by calculations that reduce the metal response to a single cavity mode and neglect self-consistency—for example, the modification of the cavity response induced by radiation from the molecule. To assess the role of this self-consistent response, we compare our results with those obtained from a simplified single–cavity-mode TERS model employed in previous studies.

As a representative simplified nanocavity model used in recent resonant single-molecule TERS analysis modeled the Raman map using a point-charge approximation of the nanocavity together with a single-plasmon-mode picture.[28] Motivated by this class of simplified treatments, we here construct an analogous non-self-consistent reference model in which the tip/substrate optical responses are represented by point dipoles and the TERS signal is evaluated without self-consistent Stokes-side backaction. In particular, we focus on the $S_2$-resonant TERS of the four-level pentacene model considered in the main text and perform the calculations accordingly. Under the resonance conditions considered in the main text, the near field at frequency $\omega_i$ is associated with the transition from state $|1\rangle$ to state $|4\rangle$, whereas the scattered field at frequency $\omega_s$ is associated with the transition from state $|4\rangle$ to state $|2\rangle$. In this calculation, the TERS intensity $I_{\mathrm{TERS}}$ is expressed as follows:

$$I_{\mathrm{TERS}} \propto |f_{14}(\omega_i)|^2 |f_{42}(\omega_s)|^2, \tag{S63}$$

$$f_{nm}(\omega) = \int d\boldsymbol{r}\, \boldsymbol{\mathcal{P}}_{nm}(\boldsymbol{r}) \cdot [\boldsymbol{G}_{\mathrm{vac}}(\boldsymbol{r}, \boldsymbol{r}_{\mathrm{tip}}, \omega) + \boldsymbol{G}_{\mathrm{vac}}(\boldsymbol{r}, \boldsymbol{r}_{\mathrm{sub}}, \omega)]\boldsymbol{p}, \tag{S64}$$

where $\boldsymbol{r}_{\mathrm{tip}}$ and $\boldsymbol{r}_{\mathrm{sub}}$ denote the positions of the point dipoles representing the tip and the substrate, respectively. As shown in Fig. S8(a), we place the point dipole representing the tip at a height $D_{\mathrm{tip}}$ above the molecular center and the point dipole representing the substrate at a depth $D_{\mathrm{sub}}$ below the molecular center, and perform calculations for several values of

$D_{\text{tip}}$ and $D_{\text{sub}}$.

Figure S8(b) reproduces the calculated $S_2$-resonant TERS map for the v5 vibrational mode of pentacene presented in the main text (full self-consistent theory). Figures S8(c)–(e) show the corresponding $S_2$-resonant TERS maps for the same v5 pathway obtained using Eq. (S63), with $(D_{\text{tip}}, D_{\text{sub}}) = (0.5\text{ nm}, 1.5\text{ nm})$, $(1.0\text{ nm}, 2.0\text{ nm})$, and $(1.5\text{ nm}, 2.5\text{ nm})$, respectively. As the distance between the point dipoles and the molecule increases, the regions of enhanced scattering progressively shift away from the molecular center.

The key point is that the self-consistent $S_2$-resonant TERS map in Fig. S8(b) differs qualitatively from the non-self-consistent results in Figs. S8(c)–(e). In the self-consistent calculation, the scattering enhancement appears at two sites separated along the molecular long axis, whereas all three non-self-consistent results exhibit four-lobe–like features. Thus, the difference is not merely a change in signal magnitude; the apparent image symmetry itself changes. This would lead to a different interpretation in a parity-based mode-discrimination scheme.

This qualitative difference can be understood as follows. Figures S9(a) and (b) schematically illustrate, for the self-consistent and non-self-consistent calculations, respectively, the interaction between the in-plane molecular polarization and the tip dipole located directly above it. In the self-consistent calculation, the molecule and the metal can interact because a dipole is induced on the tip along the direction of the electric lines of force emitted by the molecular polarization (Fig. S9(a)). In contrast, in the non-self-consistent calculation, the dipole on the metal is fixed to a direction orthogonal to those electric lines of force regardless of their orientation, and thus the molecule and the metal cannot interact properly (Fig. S9(b)). This effect contributes particularly strongly in the scattered field, where the molecule itself acts as the source of the radiation.

As a result, in the self-consistent calculation, even at positions where the interaction between the excitation-induced near field and the HT transition dipole moment is maximal, finite radiation still arises because the FC transition dipole moment—behaving as a

multipole—can interact with the tip; consequently, the structure of the HT transition dipole moment appears in the TERS map. In contrast, in the non-self-consistent calculation, the FC transition dipole moment cannot interact with the tip along the molecular long axis, causing the HT-induced contrast to cancel out in the TERS map. This is considered to be the reason why the TERS-map features differ between Fig. S8(b) and Figs. S8(c)–(e).

These results support the conclusion that a self-consistent treatment of the Stokes-side electrodynamics is essential for correctly evaluating parity-sensitive TERS maps and for transferring the symmetry information of the forbidden-resonance (HT-related) pathway into the observable Raman image.

# References


(1) Albrecht, A. C. On the Theory of Raman Intensities. *The Journal of Chemical Physics* **1961**, *34*, 1476–1484.

(2) Long, D. A. *The Raman Effect: A Unified Treatment of the Theory of Raman Scattering by Molecules*; John Wiley & Sons, Ltd., 2002.

(3) Novotny, L.; Hecht, B. *Principles of Nano-Optics*, 2nd ed.; Cambridge University Press, 2012.

(4) Carminati, R.; Greffet, J.-J. Near-Field Effects in Spatial Coherence of Thermal Sources. *Physical Review Letters* **1999**, *82*, 1660–1663.

(5) Wubs, M.; Suttorp, L. G.; Lagendijk, A. Multiple-scattering approach to interatomic interactions and superradiance in inhomogeneous dielectrics. *Phys. Rev. A* **2004**, *70*, 053823.

(6) Born, M.; Oppenheimer, R. Zur Quantentheorie der Molekeln. *Annalen der Physik* **1927**, *389*, 457–484.

(7) Tomoshige, Y.; Tamura, M.; Yokoyama, T.; Ishihara, H. Enhanced photoluminescence of strongly coupled single molecule-plasmonic nanocavity: analysis of spectral modifications using nonlocal response theory. *Nanophotonics* **2025**, *14*, 1157–1169.

(8) Goodman, J. J.; Draine, B. T.; Flatau, P. J. Application of fast-Fourier-transform techniques to the discrete-dipole approximation. *Opt. Lett.* **1991**, *16*, 1198–1200.

(9) Manian, A.; Shaw, R. A.; Lyskov, I.; Wong, W.; Russo, S. P. Modeling radiative and non-radiative pathways at both the Franck–Condon and Herzberg–Teller approximation level. *The Journal of Chemical Physics* **2021**, *155*, 054108.

(10) Shiraki, H.; Yokoshi, N.; Ishihara, H. Chirality-selective superfluorescence based on chiral interactions. *Phys. Rev. A* **2022**, *106*, 053511.

(11) Dirac, P. A. M. The quantum theory of the emission and absorption of radiation. *Proceedings of the Royal Society of London. Series A, Containing Papers of a Mathematical and Physical Character* **1927**, *114*, 243–265.

(12) Duan, S.; Tian, G.; Xie, Z.; Luo, Y. Gauge invariant theory for super high resolution Raman images. *The Journal of Chemical Physics* **2017**, *146*, 194106.

(13) Kamandar Dezfouli, M.; Hughes, S. Quantum Optics Model of Surface-Enhanced Raman Spectroscopy for Arbitrarily Shaped Plasmonic Resonators. *ACS Photonics* **2017**, *4*, 1245–1256.

(14) Zhang, Y.; Dong, Z.-C.; Aizpurua, J. Theoretical treatment of single-molecule scanning Raman picoscopy in strongly inhomogeneous near fields. *Journal of Raman Spectroscopy* **2021**, *52*, 296–309.

(15) Freedman, T. B.; Gao, X.; Shih, M.-L.; Nafie, L. A. Electron Transition Current Density in Molecules. 2. Ab Initio Calculations for Electronic Transitions in Ethylene and Formaldehyde. *The Journal of Physical Chemistry A* **1998**, *102*, 3352–3357.

(16) Macak, P.; Luo, Y.; Ågren, H. Simulations of vibronic profiles in two-photon absorption. *Chemical Physics Letters* **2000**, *330*, 447–456.

(17) Domcke, W.; Cederbaum, L. S. Theory of the vibrational structure of resonances in electron-molecule scattering. *Phys. Rev. A* **1977**, *16*, 1465–1482.

(18) Cerezo, J.; Santoro, F. FCclasses3: Vibrationally-resolved spectra simulated at the edge of the harmonic approximation. *Journal of Computational Chemistry* **2023**, *44*, 626–643.

(19) Xu, J.; Zhu, X.; Tan, S.; Zhang, Y.; Li, B.; Tian, Y.; Shan, H.; Cui, X.; Zhao, A.; Dong, Z.; Yang, J.; Luo, Y.; Wang, B.; Hou, J. G. Determining structural and chemical heterogeneities of surface species at the single-bond limit. *Science* **2021**, *371*, 818–822.

(20) Michalska, D.; Wysokiński, R. The prediction of Raman spectra of platinum (II) anticancer drugs by density functional theory. *Chemical Physics Letters* **2005**, *403*, 211–217.

(21) Frisch, M. J. et al. Gaussian16 Revision C.01. 2016; Gaussian Inc. Wallingford CT.

(22) Becke, A. D. Density‐functional thermochemistry. III. The role of exact exchange. *The Journal of Chemical Physics* **1993**, *98*, 5648–5652.

(23) Hariharan, P. C.; Pople, J. A. The influence of polarization functions on molecular orbital hydrogenation energies. *Theoretica chimica acta* **1973**, *28*, 213–222.

(24) Hirata, S.; Head-Gordon, M. Time-dependent density functional theory within the Tamm–Dancoff approximation. *Chemical Physics Letters* **1999**, *314*, 291–299.

(25) Vial, A.; Laroche, T. Comparison of gold and silver dispersion laws suitable for FDTD simulations. *Appl. Phys. B* **2008**, *93*, 139–143.

(26) Zhang, R.; Zhang, Y.; Dong, Z. C.; Jiang, S.; Zhang, C.; Chen, L. G.; Zhang, L.; Liao, Y.; Aizpurua, J.; Luo, Y.; Yang, J. L.; Hou, J. G. Chemical mapping of a single molecule by plasmon-enhanced Raman scattering. *Nature* **2013**, *498*, 82–86.

(27) Yamanishi, J.; Yamane, H.; Naitoh, Y.; Li, Y. J.; Yokoshi, N.; Kameyama, T.; Koyama, S.; Torimoto, T.; Ishihara, H.; Sugawara, Y. Optical force mapping at the single-nanometre scale. *Nature Communications* **2021**, *12*, 3865.

(28) de Campos Ferreira, R. C.; Sagwal, A.; Dolezal, J.; Canola, S.; Merino, P.; Neuman, T.; Svec, M. Resonant tip-enhanced Raman spectroscopy of a single-molecule Kondo system. *Acs Nano* **2024**, *18*, 13164–13170.
